# Nanotube patterning reduces macrophage inflammatory response via nuclear mechanotransduction

**DOI:** 10.1186/s12951-023-01912-4

**Published:** 2023-07-19

**Authors:** Yiru Fu, Zheng Jing, Tao Chen, Xinxin Xu, Xu Wang, Mingxing Ren, Yanqiu Wu, Tianli Wu, Yuzhou Li, He Zhang, Ping Ji, Sheng Yang

**Affiliations:** 1 College of Stomatology, Chongqing Medical University, 426# Songshi-bei Road, Yubei District, Chongqing, 401147 China; 2 Chongqing Key Laboratory of Oral Diseases and Biomedical Sciences, Chongqing, China; 3 Chongqing Municipal Key Laboratory of Oral Biomedical Engineering of Higher Education, Chongqing, China

**Keywords:** Titania nanotube patterning, Anodization, Mechanotransduction, Macrophage, Inflammatory response

## Abstract

The inflammatory immune environment surrounding titanium bone implants determines the formation of osseointegration, and nanopatterning on implant surfaces modulates the immune microenvironment in the implant region. Among many related mechanisms, the mechanism by which nanopatterning controls macrophage inflammatory response still needs to be elucidated. In this paper, we found that inhibition of the nuclear envelope protein lamin A/C by titania nanotubes (TNTs) reduced the macrophage inflammatory response. Knockdown of lamin A/C reduced macrophage inflammatory marker expression, while overexpression of lamin A/C significantly elevated inflammatory marker expression. We further found that suppression of lamin A/C by TNTs limited actin polymerization, thereby reducing the nuclear translocation of the actin-dependent transcriptional cofactor MRTF-A, which subsequently reduced the inflammatory response. In addition, emerin, which is a key link between lamin A/C and actin, was delocalized from the nucleus in response to mechanical stimulation by TNTs, resulting in reduced actin organization. Under inflammatory conditions, TNTs exerted favourable osteoimmunomodulatory effects on the osteogenic differentiation of mouse bone marrow-derived stem cells (mBMSCs) in vitro and osseointegration in vivo. This study shows and confirms for the first time that lamin A/C-mediated nuclear mechanotransduction controls macrophage inflammatory response, and this study provides a theoretical basis for the future design of immunomodulatory nanomorphologies on the surface of metallic bone implants.

**Supplementary Information:**

The online version contains supplementary material available at 10.1186/s12951-023-01912-4.

## Introduction

Osteoimmunomodulation orchestrates the process of osseointegration by mediating immune cell responses and subsequent bone cell behaviours [1, 2]. Within hours after implantation, the host innate immune system is activated at the interface of the implant, and the first stage of bone healing, namely, the inflammatory stage, begins [3, 4]. The normal inflammatory process establishes a proregenerative environment that facilitates osseointegration [5, 6]. However, a large number of patients who receive endosseous implantation suffer from chronic diseases that hamper osseointegration, for example, diabetes, aging, and aging-related diseases such as osteoporosis. In these pathophysiological situations, a common feature is sustained proinflammatory systemic state [7–10]. This dysregulated inflammatory microenvironment inhibits regenerative cell infiltration and differentiation, leading to poor osseointegration [11, 12]. Thus, it is important to maintain proper inflammatory conditions to promote optimal osseointegration, and implanted biomaterials with immunomodulatory properties could be a viable strategy for controlling inflammation and promoting healing [13, 14].

Tuning the nanopatterning of implant surfaces is an efficient strategy to manipulate osteoimmunomodulation to promote bone tissue regeneration [15, 16]. Nanopatterning mimics the natural nanostructures of bone and is highly tuneable; thus, nanopatterning has the potential to precisely manipulate osteoimmune responses [17]. Among all immune cells, macrophages are one of the most important types of effector cells that are responsible for bone regeneration [18, 19], and macrophages exhibit high plasticity in response to physical stimulation [20, 21]. Previous studies have shown that nanotopography influences macrophage morphology to regulate cell function and outputs [22, 23]. Titania nanotubes inhibit the adhesion and integrin expression of macrophages with downregulation of inflammatory proteins MCP-1, IL-6, and IL-8 [17]. Nanopits suppress actin polymerization with impaired nuclear translocation of myocardin-related transcription factor-A (MRTF-A), leading to the downregulation of the inflammatory genes IL-1β, IL-6, and CXCL9 [24]. Although these studies preliminarily elucidated that nanopatterning modulates macrophage response by changing cellular adhesion and cytoskeleton structure, the underlying mechanisms are still not fully understood.

Emerging evidence indicates that nuclear mechanotransduction plays a crucial role in sensing surface nanopatterning and regulating cell fate [25–27]. Specifically, nucleoskeleton lamin A/C, which is a protein meshwork under the nuclear membrane, is dynamically remodelled in response to mechanical stimulation [28, 29], and it participates in the developmental processes of numerous cell types, including immune cells [30–32]. On the one hand, lamin A/C expression is increased in cells that are grown on stiff matrices, thus enhancing stem cell osteogenic differentiation, and these results indicate the important role of lamin A/C in mediating the behaviour of cells that are seeded on biomaterial substrates [28, 33, 34]. On the other hand, lamin A/C contributes to the differentiation and activation of immune cells, such as T cells [35, 36] and macrophages [37], and depletion of lamin A/C results in a significant downregulation of relevant target genes. Therefore, we hypothesized that lamin A/C may participate in nanopatterning-mediated macrophage regulation.

As TNTs have demonstrated favourable osteo-immune capacity in bone regeneration in healthy conditions [38], we supposed whether TNTs could be still capable of modulating macrophage inflammatory response under systemic inflammation that may happen in certain patients suffering from chronic conditions. Here, we found that nanotubes significantly decreased the lipopolysaccharide (LPS)-induced inflammatory response of RAW264.7 cells and suppressed lamin A/C expression. Moreover, the role of lamin A/C in the nanostructure-mediated regulation of macrophage inflammatory response was elucidated by overexpressing or silencing lamin A/C in macrophages and measuring inflammatory signalling factors levels. Finally, the immunomodulatory effect of nanotube structures on osteogenesis under chronic inflammation was investigated in vitro and in vivo. This study is expected to deepen the understanding of the mechanism by which nanopatterning exerts immunomodulatory effects and to lay a theoretical foundation for the development of novel immunomodulatory biomaterials.

## Materials and methods

### Titanium sample preparation and surface characterization

Pure Ti discs (purity: 99.9%, diameter: 34 or 14 mm, thickness: 1 mm, Baoji Titanium Industry, China) were burnished from #320 to #2000 with silicon carbide sandpaper and were ultrasonically cleaned to generate the smooth titanium surface (pTi). Titania nanotubes of different sizes were fabricated according to the methods described in our previous studies [39, 40]. Briefly, polished Ti was anodized in an aqueous electrolyte (5 g NH_4_F dissolved in a mixed solution of 500 ml deionized water and 500 ml glycerol) at various voltages (10, 20, and 30 V) for 1 h and then annealed at 450 °C for 1 h. All the samples were sterilized using 75% ethanol and ultraviolet irradiation before cell culture. The surface topography of the titanium samples was observed via field emission scanning electron microscopy (SEM, Hitachi SU8010, Japan).

### Cell culture

The RAW264.7 macrophage cell line was purchased from the American Type Culture Collection (ATCC, USA). RAW264.7 cells were maintained in Dulbecco’s modified Eagle’s medium (DMEM, Gibco, USA) supplemented with 10% foetal bovine serum (FBS, Gibco, USA) and 1% penicillin‒streptomycin solution (Solarbio, China) in a humidified atmosphere of 5% CO_2_ at 37℃. mBMSCs were isolated from the femur bones of C57BL/6 mice (6–8 weeks, male) according to a previously described protocol [41].

### Drug treatment

Proinflammatory activation was achieved by stimulating RAW264.7 cells with LPS (25 ng/ml) (Solaria, China) for 6 h. For cytoskeleton-related studies, the cells were pretreated with cytoskeleton inhibitors (cytochalasin D (4 µM, potent inhibitor of actin polymerization) and blebbistatin (50 µM, potent inhibitor of myosin II motor activity)) for 1 h prior to LPS stimulation for 6 h.

### Quantitative real-time PCR (qRT‒PCR)

Total cellular RNA was isolated by Trizol reagent (Takara, Japan) and reverse-transcribed into cDNA using PrimeScript Master Mix (Takara, Japan). Quantitative real-time PCR was performed with TB Green Premix Ex Taq II (Takara, Japan) in a CFX96 Real-Time PCR Detection System (Bio-Rad, USA). The primer sequences specific for the target genes are listed in Table S1, and glyceraldehyde-3-phosphate dehydrogenase (GAPDH) served as the reference gene. CXCL9, as a representative gene marker for late LPS-activated inflammatory response.

### Enzyme-linked immunosorbent assay (ELISA)

RAW264.7 cells were seeded on pTi and TNTs and incubated for 24 h, and then, the cells were stimulated with LPS for 6 h. Then, the cell culture medium was collected, and the concentrations of IL-6, IL-1β, and CXCL9 were measured using ELISA kits following the manufacturer’s protocol (RUIXIN BIOTECH, China).

### Morphological observation of macrophages

The samples were fixed in 2.5% glutaraldehyde for 30 min and then dehydrated with 30%, 50%, 70%, 90%, and 100% ethanol solutions for 10 min each. Finally, cell morphology was observed by SEM.

### Western blotting

Whole-cell lysates were obtained using RIPA lysis buffer (Beyotime, China) supplemented with protease and phosphatase inhibitors (Sigma, USA). Cytosolic and nuclear fractions were extracted using the Nuclear and Cytoplasmic Protein Extraction Kit (Beyotime, China). The proteins were separated by sodium dodecyl sulfate‒polyacrylamide gel electrophoresis (SDS‒PAGE) and transferred to polyvinylidene fluoride (PVDF) membranes (pore diameter = 0.22 μm). After being incubated in 5% skim milk (Sigma, USA) for 2 h at room temperature (RT), the bands were incubated overnight at 4℃ with primary antibodies. Then, the bands were incubated with HRP-conjugated secondary antibodies for 2 h at RT and imaged with a chemiluminescent reagent (Merck Millipore, USA) in an imaging system (Bio-Rad, USA). The Western blotting results were analysed using ImageJ software. All primary antibodies and secondary antibodies used in Western blotting are listed in table S2.

### Transfection

For lamin A/C overexpression, RAW264.7 cells were transfected with lentiviral pEZ-Lv105-Lmna to generate lamin A/C-overexpressing cells. For lamin A/C or emerin knockdown, RAW264.7 cells were transfected siRNA using PepMute siRNA Transfection Reagent (SignaGen, USA). The siRNA sequences were as follows: lamin A/C siRNA (GACGAUCCUUUGAUGACC-UAU) and emerin siRNA smart pool (GACCUCACU-UGUAGAUGCU, CUCACUUCA-UUAGAGGAAA, CCUGUUU-GUUGUCACCU-UU). The efficiency of target protein overexpression or knockdown was verified by Western blotting experiments.

### Immunofluorescence staining

Cells were fixed in 4% paraformaldehyde (Sigma) for 10 min and permeabilized with 0.1% Triton X-100 (Sigma) for 5 min. Next, the cells were incubated with primary antibodies at 4 °C overnight and then incubated with Alexa Fluor-conjugated secondary antibodies (Invitrogen, USA) (1:300) and DAPI (Beyotime, China) (1:1000). Filamentous actin (F-actin) was labelled using Alexa Fluor 647-conjugated phalloidin (Invitrogen, USA), and globular actin (G-actin) was labelled using Alexa Fluor 488-conjugated DNase-I (Invitrogen, USA). Immunofluorescence images were obtained by laser scanning confocal microscopy (LSCM, Germany) and quantitatively analysed using CellProfiler and ImageJ. All primary antibodies and secondary antibodies used in immunostaining are listed in table S3.

### Effect of nanopatterning-mediated osteoimmunity on osteogenesis

RAW264.7 cells were seeded on pTi and TNTs surfaces, incubated for 24 h, and stimulated with LPS for 6 h. Then, the supernatants of each group were collected and diluted 1:3 in osteogenic medium (OM), which consisted of α-Minimal Essential Medium (α-MEM, Sigma, USA), 10% FBS, 10 mM β-glycerophosphate (Sigma, USA), 50 µg/ml ascorbic acid (Sigma, USA) and 10 nM dexamethasone (Sigma, USA). To investigate whether nanopatterning affected the osteogenic differentiation of mBMSCs by mediating the macrophage-related immune microenvironment, mBMSCs were seeded on tissue culture polystyrene (TCPS) and cultured in different macrophage conditional media. To further explore the crosstalk between macrophages and mBMSCs on different nanotopographies, mBMSCs were seeded on pTi and TNTs surfaces and cultured with conditioned medium corresponding to the surface. All the samples were cultured for 7 days to measure the expression of osteogenic genes using qRT‒PCR and for 21 days to analyse extracellular matrix mineralization using Alizarin Red S (ARS) staining (ARS, Solarbio, China).

### Animal study

All the animal experiments were performed according to the guidelines of the Animal Care and Use Committee of China and were approved by the ethics committee of Chongqing Medical University Affiliated Hospital of Stomatology (Ethics No. CQHS-REC-2022 (LSNo.093)). Sprague‒Dawley (SD) rats (male, 250–300 g) were divided into three groups (n = 6 rats per group) as follows: (1) pTi_PBS_ group (implantation of pTi rods with PBS administration), (2) pTi group (implantation of pTi rods with LPS administration), and (3) TNTs group (implantation of TNTs rod with LPS administration). After intraperitoneal injection of 5% chloral hydrate (0.7 ml/100 g) for anaesthesia, the rat distal femurs were surgically exposed, implanted with a prepared Ti rod (diameter: 1 mm, length: 10 mm, Baoji titanium Industry, China), and sutured in layers using 6 − 0 silk braided sutures. According to previous studies [42, 43], the rats were intraperitoneally injected with LPS (5 mg/kg, dissolved in PBS) on days 1, 5, and 9 to induce chronic inflammation, and phosphate buffer saline (PBS) was used as a control. All the rats were sacrificed by CO_2_ hypoxia on day 14 postoperation.

### Osseointegration evaluation

All the samples were scanned with micro-CT (vivaCT80, SCANCO Medical AG, Switzerland). A ring radius of 300 μm surrounding the implant was defined as the region of interest (ROI). Three-dimensional images, bone volume per tissue volume (BV/TV), and trabecular thickness (Tb. Th) were analysed using SCANCO VivaCT40 micro-CT software. Then, the samples were fixed in 4% paraformaldehyde, decalcified with Na-EDTA (Servicebio, China) for 40 days, embedded in paraffin, and cut into sections (thickness: 7 μm). Haematoxylin-eosin (H&E) staining (Solarbio, China) was performed with commercial kits following the manufacturer’s instructions.

### Statistical analysis

All the results are expressed as the mean ± standard deviation. Statistical analysis of differences between two groups was evaluated with unpaired Student’s t test. Statistical analysis of differences among three or more groups was evaluated with a one-way analysis of variance. p < 0.05 was considered to indicate statistical significance. GraphPad Prism 9 (GraphPad, USA) software was used for all the statistical analyses and graphical representations.

## Results

### TNTs downsized the inflammatory response of macrophages

**Fig. 1:**
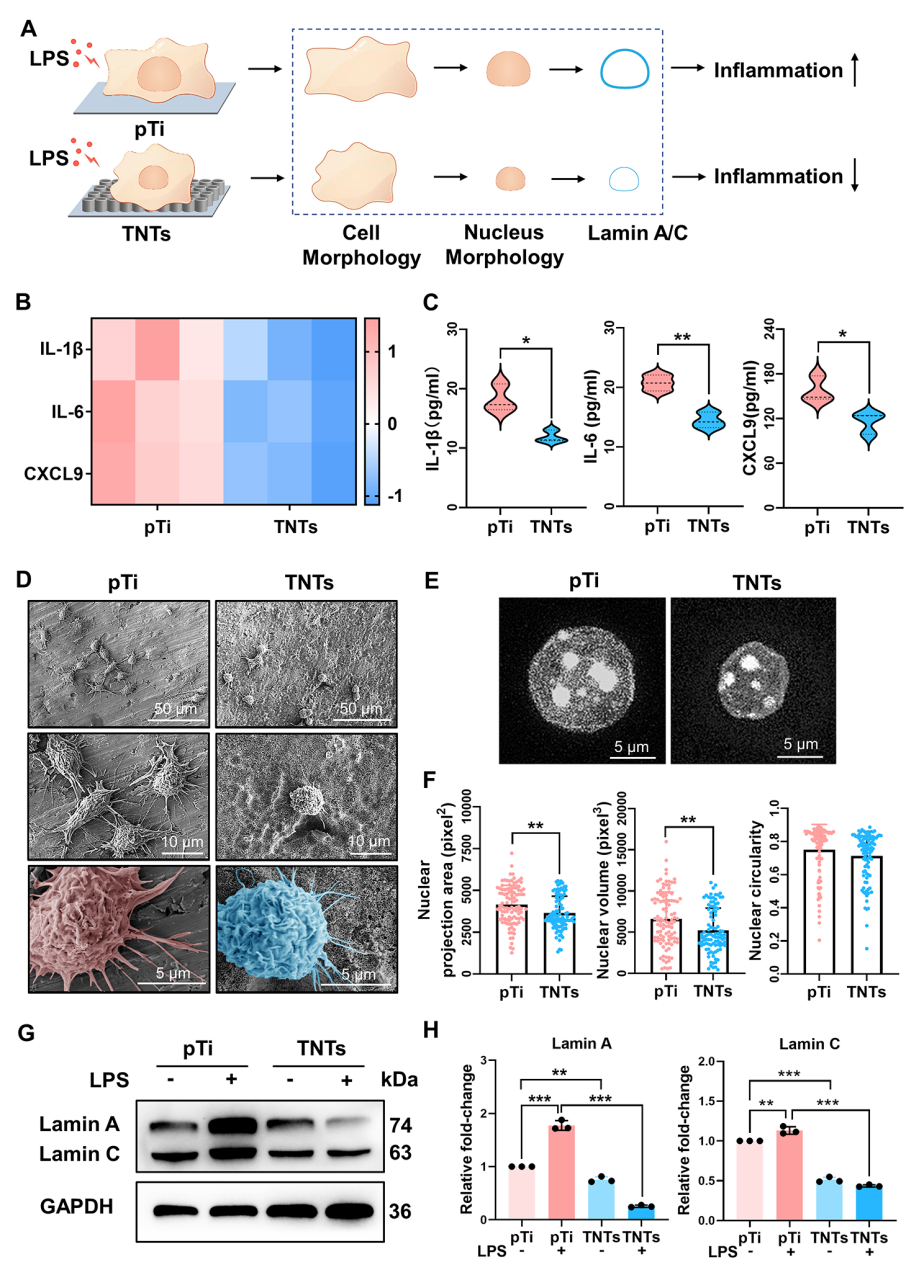
**Nanotube patterning inhibited macrophage inflammatory response and nucleoskeleton lamin A/C expression. (A)** Schematic illustration showing that TNTs modulated macrophage inflammatory response, which was accompanied by changes in macrophage morphology, nuclear shape and lamin A/C levels. B-C) Heatmap of relative inflammatory gene expression **(B)** and cytokine secretion **(C)** of macrophages cultured on pTi and TNTs with 6 h LPS treatment. **(D)** Cell morphologies of LPS-treated macrophages cultured on pTi and TNTs; scale bars, 50 μm (first panel), 10 μm (second panel) and 5 μm (third panel). E-F) Representative images of nuclei in RAW264.7 cells cultured on pTi and TNTs and treated with LPS **(E)** and quantitative analysis of nuclear projection area, nuclear volume, and nuclear circularity **(F)**. Data are pooled from 98–104 single cells in at least 21–30 randomly chosen fields. G-H) Lamin A/C expression in macrophages cultured on pTi and TNTs and stimulated with LPS. For qRT‒PCR, ELISA, and Western blotting, quantification data are shown as averages of three biological replicates. All the values are mean ± SD. *p < 0.05, **p < 0.01, ***p < 0.001

Titania nanotubes of different sizes were synthesized by the anodic oxidation method and characterized by SEM (Fig. S1). All the titania nanotube surfaces showed regular tubular structures at high magnification (scale bar: 300 nm), and the nanotube size increased with increasing voltage gradient. To investigate the immunoregulatory effect of nanotube patterning on the inflammatory response, RAW264.7 cells were seeded on different titania nanotubes, and smooth titanium (pTi) served as the control; then, the cells were stimulated with LPS. Compared with pTi, nanotube patterning exerted a substantial inhibitory effect on inflammatory gene expression, and nanotubes prepared at 30 V showed the best performance; therefore, nanotubes prepared at 30 V were selected for the following research and are henceforth referred to as TNTs (Fig. S2, Fig. 1B). Furthermore, we examined whether regulation by TNTs occurs at the cytokines secretion by ELISA, and the results (Fig. 1C) showed a significant reduction in the levels of proinflammatory cytokines, including IL-6, IL-1β and CXCL9, in the macrophages in the TNTs group compared with those in the pTi group. Collectively, these data suggested that TNTs effectively inhibited the inflammatory response of macrophages compared with pTi.

**Schema:**
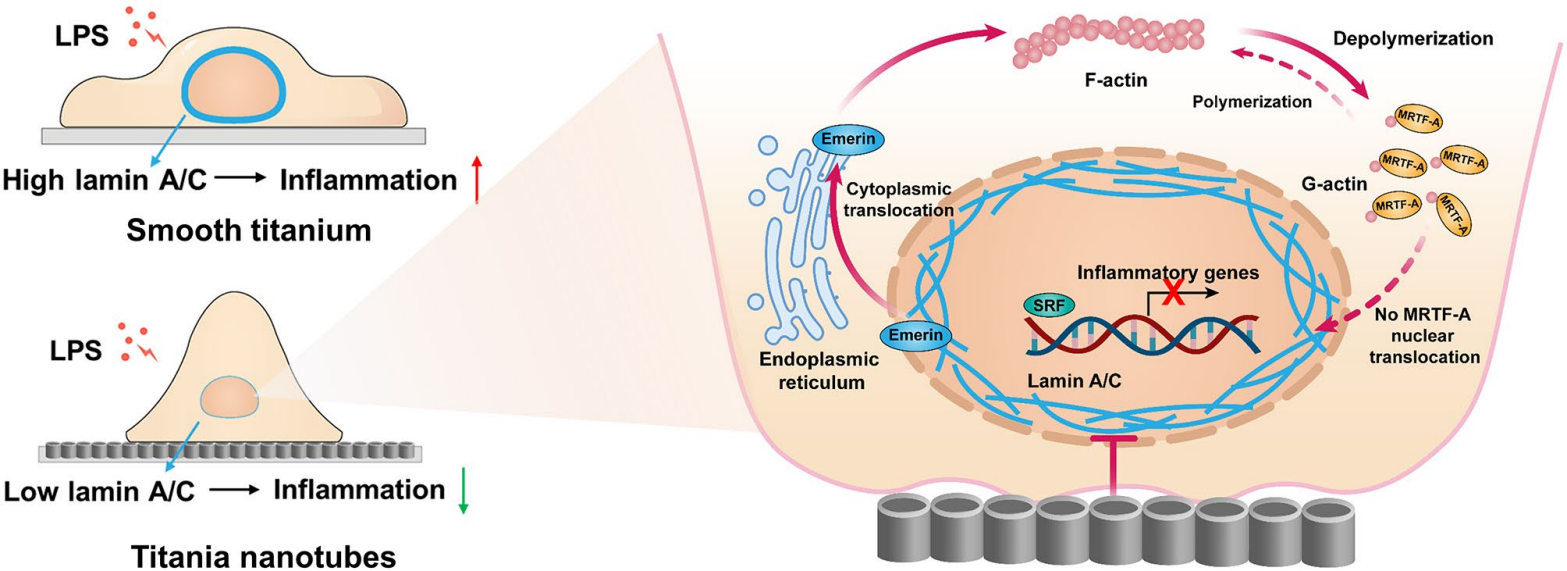
Schematic illustration of titania nanotubes regulating Lamin A/C expression to modulate macrophage inflammatory response

### TNTs caused changes in nuclear morphology and nucleoskeleton lamin A/C

To explore why LPS-treated macrophages exhibited different inflammatory responses when cultured on pTi and TNTs, we initially investigated the underlying mechanism by observing cell morphology. As shown in Fig. 1D, RAW264.7 cells on pTi were fully spread, with abundant filopodia stretching across long distances. In contrast, macrophages on TNTs contracted and formed round spheres with sparse filopodia. Consistently, according to DAPI and phalloidin staining (Fig. S3), RAW264.7 cells seeded on pTi exhibited polygonal shapes and spread, whereas cells seeded on TNTs were spherical and nonspreading. Quantitatively, the cytoplasmic area of the macrophages seeded on TNTs was significantly lower than that in the pTi group. Since cell morphology and nuclear shape are highly correlated [44, 45], we further evaluated the morphological characteristics of the nuclei of macrophages seeded on different substrates. The results (Fig. 1E, F) showed that macrophages seeded on TNTs demonstrated significantly lower nuclear projection areas and nuclear volumes than those seeded on pTi, while the nuclear circularity remained unchanged. Nucleoskeleton lamin A/C has been reported to be involved in maintaining nuclear structure [46] and mediating macrophage inflammatory response [37], so we next examined lamin A/C. After 1 h of stimulation with LPS, lamin A/C was substantially increased in cells cultured on pTi (Fig. S4). In contrast, the macrophages cultured on TNTs showed lower lamin A/C levels before and after LPS stimulation than cells cultured on pTi (Fig. 1G, H). These data indicated that lamin A/C expression was mostly affected by the inflammatory trigger LPS and its expression was notably regulated by the adhesive microenvironment. Therefore, we hypothesized that lamin A/C could play a crucial role in the regulation of macrophage inflammatory activation by nanotube patterning.

### TNTs inhibited Lamin A/C-regulated inflammation

**Fig. 2:**
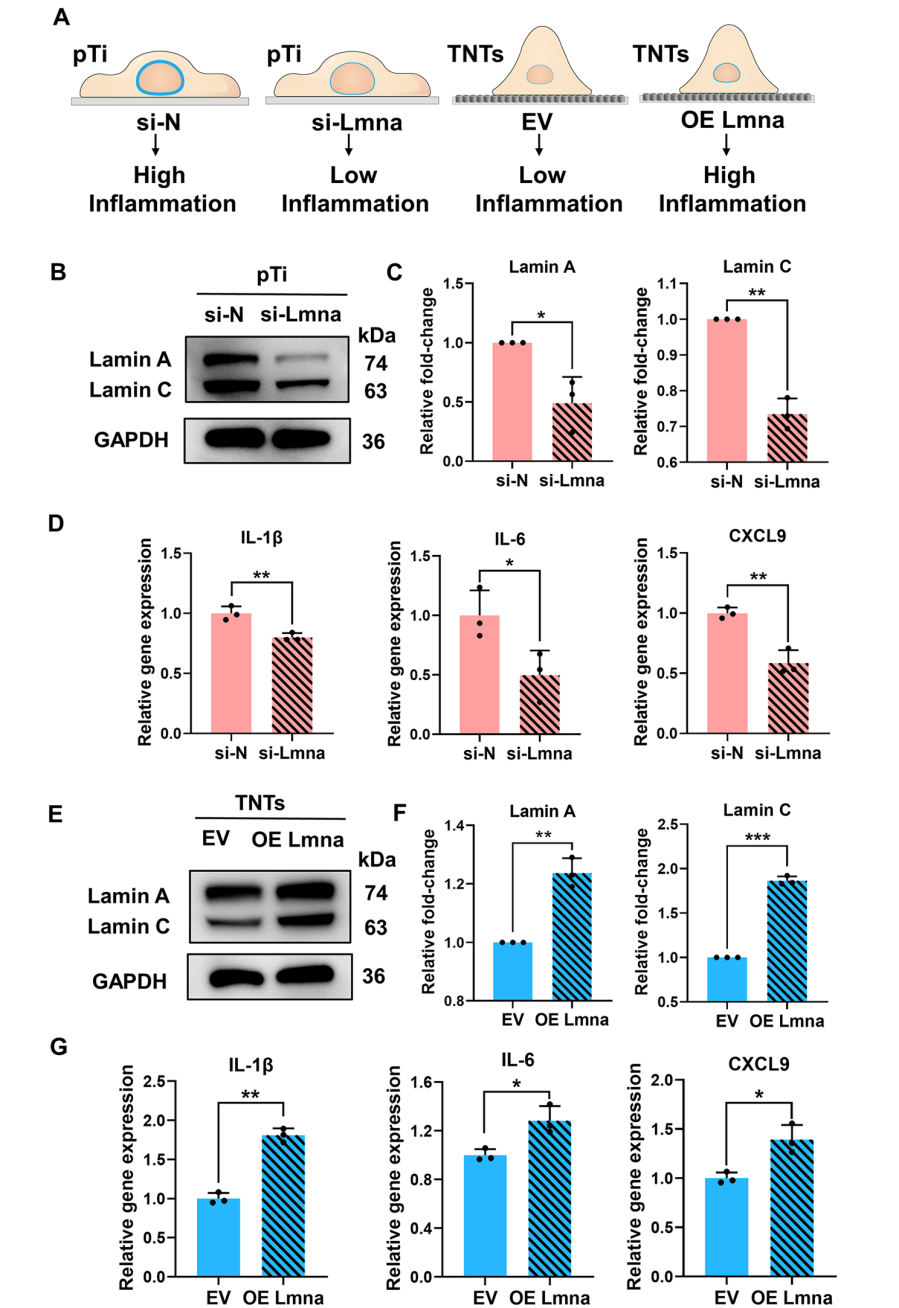
**TNTs inhibited Lamin A/C-regulated inflammation. (A)** Schematic illustration of the modulation of lamin A/C expression to regulate macrophage inflammation. B, C) Western blotting analysis of lamin A/C expression in macrophages cultured on pTi and treated with Lmna siRNA (si-Lmna) or nontargeting siRNA (si-N) for 24 h **(B)** and quantitative analysis **(C)**. **(D)** Relative expression of the inflammatory genes IL-1β, IL-6 and CXCL9 in macrophages cultured on pTi after lamin A/C knockdown and stimulation with LPS for 6 h. E, F) Western blotting analysis of lamin A/C expression in lamin A/C-overexpressing RAW264.7 or blank lentiviral vector-expressing control cells cultured on TNTs **(E)** and quantitative analysis **(F)**. G) Relative expression of the inflammatory genes IL-1β, IL-6 and CXCL9 in macrophages shown in **(E)** and stimulated with LPS. The data are presented as the mean ± SD of three biological replicates. *p < 0.05, **p < 0.01, ***p < 0.001

Our data indicated that macrophage inflammation depended on lamin A/C levels and that TNTs inhibited lamin A/C expression, suppressing macrophage inflammation. To test this hypothesis, we explored whether knockdown or overexpression of lamin A/C changed macrophage inflammatory response. Knockdown of lamin A/C in pTi-cultured macrophages with small interfering RNA (siRNA) significantly reduced lamin A/C protein levels compared with control cells that were treated with a nontargeting siRNA sequence (Fig. S5 and Fig. 2B, C). siRNA-treated macrophages that were cultured on pTi and stimulated with LPS had reduced expression of the proinflammatory genes IL-6, IL-1β and CXCL9 compared to macrophages that were treated with nontargeting control (Fig. 2D). This indicated that lamin A/C regulated the macrophage inflammatory response to LPS, and decreased lamin A/C levels were associated with lower levels of inflammation. Furthermore, we successfully constructed lamin A/C-overexpressing RAW264.7 cells via lentivirus transfection (Fig. S6). Lamin A/C-overexpressing cells cultured on TNTs showed enhanced lamin A/C protein levels compared to cells transduced with a control vector (Fig. 2E, F). LPS induced notably higher transcription of inflammation genes in lamin A/C-overexpressing macrophages cultured on TNTs than in control cells (Fig. 2G). Taken together, these data proved that TNTs tuned the macrophage inflammatory response by reducing lamin A/C levels.

### Lamin A/C modulated actin-regulated inflammation

**Fig. 3:**
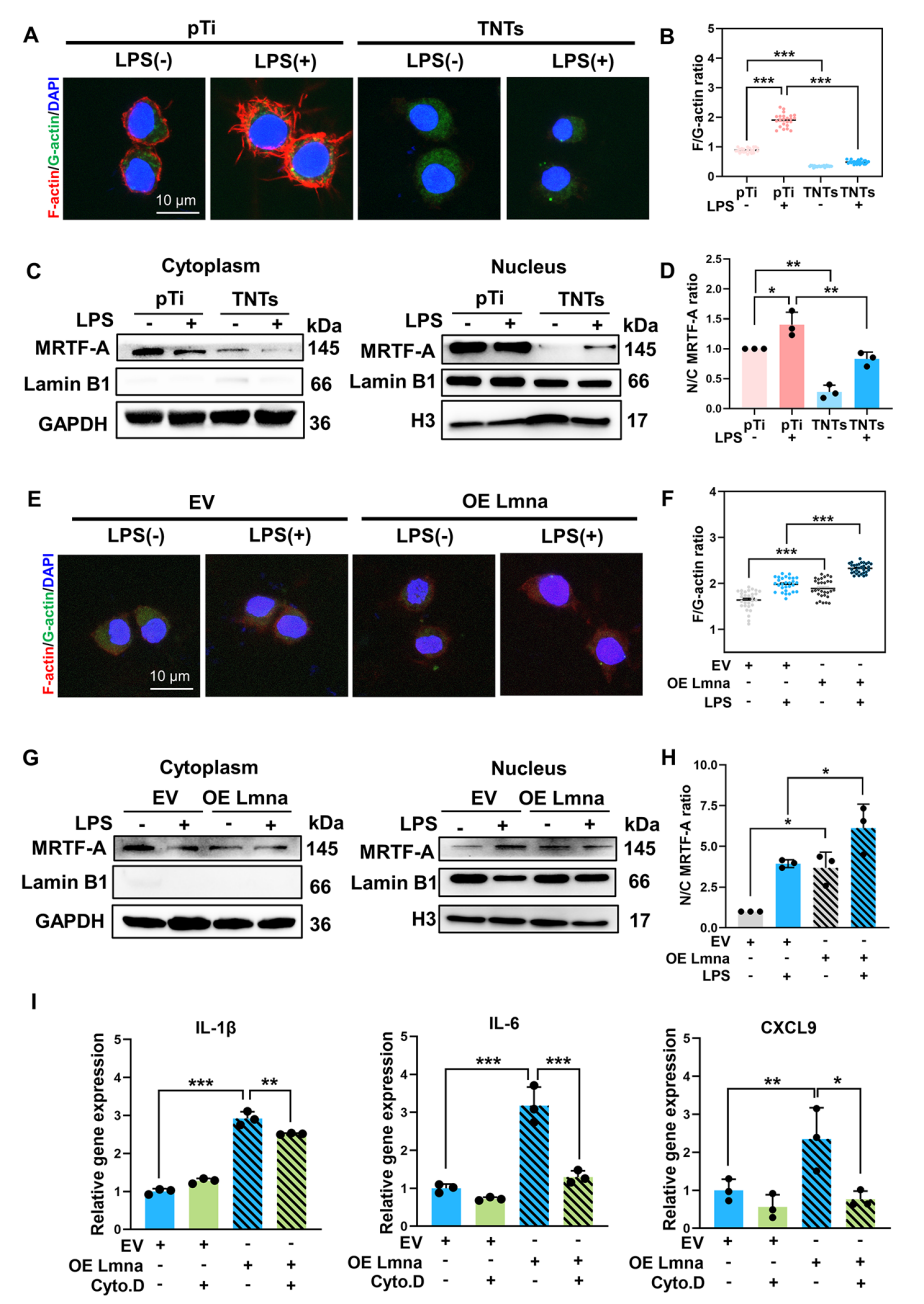
**Lamin A/C modulated actin-regulated inflammation.** A-B) Representative images and quantitative analysis of F-actin and G-actin levels in RAW264.7 cells cultured on pTi and TNTs before and after 6 h of LPS stimulation. C-D) Subcellular localization of MRTF-A and quantification of the nuclear-to-cytoplasmic ratio in RAW264.7 cells cultured on pTi and TNTs before and after LPS treatment. Lamin B1 was used as an indicator of cytoplasm-nucleus separation. H3 was the loading control for the nuclear fraction, and GAPDH was the loading control for the cytoplasmic fraction. E-F) Representative images and quantitative analysis of F-actin and G-actin levels in lamin A/C-overexpressing macrophages cultured on TNTs and stimulated with LPS. G-H) Subcellular localization of MRTF-A and quantification of the nuclear-to-cytoplasmic ratio in lamin A/C-overexpressing macrophages cultured on TNTs and stimulated with LPS. I) Inflammatory gene expression in lamin A/C-overexpressing and cytochalasin D (Cyto. D) + lamin A/C-overexpressing macrophages after LPS stimulation. Quantification of the F/G-actin ratio was performed on pooled samples from 23–31 single cells in each group from at least 8–12 randomly chosen fields. Western blotting and qRT‒PCR data are presented as the mean ± SD of three biological replicates. *p < 0.05, **p < 0.01, ***p < 0.001

Previous studies have shown that LPS stimulates actin polymerization, followed by downstream MRTF-A translocation into the nucleus, which drives the expression of proinflammatory genes [24]. As lamin A/C regulates actin dynamics [47], we next investigated whether lamin A/C-mediated macrophage inflammatory response were dependent on actin polymerization. After LPS stimulation, the F/G-actin ratio significantly ramped up which was accompanied by increased nuclear translocation of MRTF-A in macrophages cultured on pTi (Fig. 3A-D). In contrast to the pTi group, the F/G-actin ratio in the TNTs group was downsized before and after LPS stimulation, and the nuclear translocation of MRTF-A was also decreased, indicating that the actin-regulated inflammatory pathway was suppressed by adhesion to TNTs. In addition, lamin A/C-overexpressing cells cultured on TNTs showed a higher F/G-actin ratio and greater nuclear translocation of MRTF-A than control cells before and after LPS treatment (Fig. 3E-H). To evaluate whether lamin A/C-mediated regulation of inflammatory gene expression in macrophages cultured on TNTs indeed depended on actin polymerization, lamin A/C-overexpressing macrophages were seeded on TNTs, pretreated with cytochalasin D (inhibitor of actin polymerization), and stimulated with LPS. The results (Fig. 3I) showed that the lamin A/C overexpression-induced upregulation of inflammatory markers in macrophages cultured on TNTs was significantly suppressed by cytochalasin D, suggesting that lamin A/C-mediated regulation of inflammation in macrophages cultured on TNTs depends on the actin-regulated MRTF-A-SRF pathway.

### Emerin was required for the modulation of inflammation by Lamin A/C

**Fig. 4:**
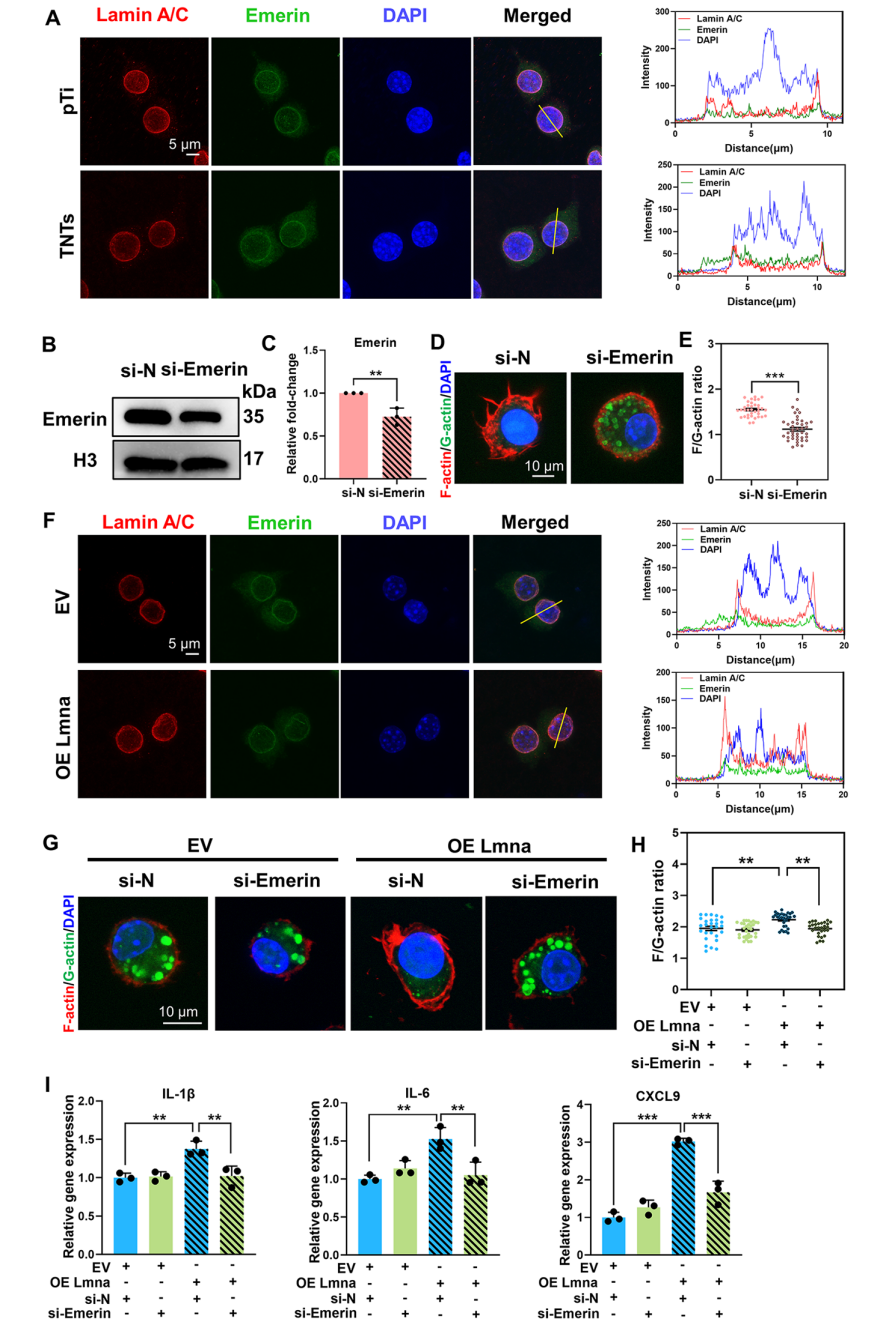
**Emerin was required for the modulation of inflammation by Lamin A/C.** A) Representative images of emerin in macrophages cultured on pTi and TNTs and stimulated with LPS for 6 h. Right: normalized fluorescence intensity profiles of lamin A/C (red), emerin (green) and DAPI (blue) along the yellow line crossing cells are shown. Scale bar, 5 μm. B-C) Effects of emerin siRNA (si-emerin) or negative control siRNA (si-N) transfection on RAW264.7 cells cultured on pTi were analysed by western blotting. D-E) Representative images of the F/G-actin ratio in emerin siRNA-transfected macrophages cultured on pTi and stimulated with LPS. F) Representative images of emerin in lamin A/C-overexpressing macrophages cultured on TNTs after 6 h of LPS stimulation. Representative images of F/G-actin (G-H) and inflammatory gene expression **(I)** in lamin A/C-overexpressing macrophages and si-emerin + lamin A/C-overexpressing macrophages cultured on TNTs after LPS stimulation. The data for analysing the F/G-actin ratio were pooled from 28–40 single cells in each group from at least 6–10 randomly chosen fields. Western blotting and qRT‒PCR data are presented as the mean ± SD of three biological replicates. *p < 0.05, **p < 0.01, ***p < 0.001

**Fig. 5:**
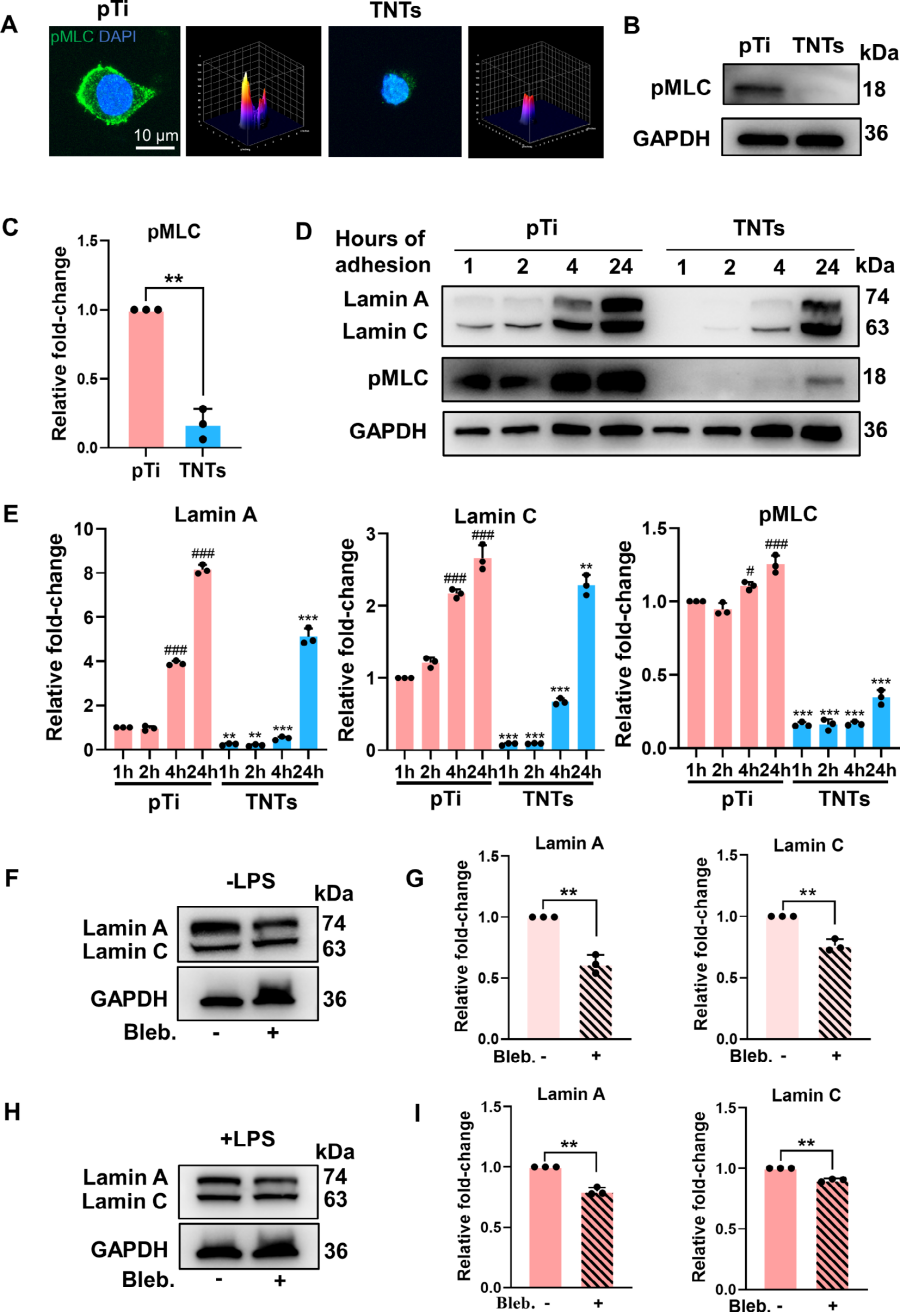
**TNTs regulated lamin A/C levels via actomyosin contractility.** A-C) Representative fluorescence images with corresponding 3D surface plot images **(A)** and Western blotting analysis (B, C) of phosphorylated myosin light chain (pMLC) levels in macrophages cultured on pTi and TNTs and stimulated with LPS for 6 h. D, E) Western blotting analysis of pMLC and lamin A/C levels in RAW264.7 cells cultured on pTi and TNTs for 1, 2, 4, and 24 h. F, G) Western blotting analysis of lamin A/C in macrophages cultured on pTi and treated with blebbistatin (Bleb.). H, I) Under LPS stimulation, the levels of lamin A/C in macrophages cultured on pTi and treated with blebbistatin. The data are presented as the mean ± SD of three biological replicates. * When comparing TNTs to pTi or # when comparing 4 or 24 h versus 1 h of adhesion, *p < 0.05, **p < 0.01, ***p < 0.001

Nuclear lamina-associated lamin A/C determines the localization of emerin, which is an inner nuclear membrane protein [48]. When lamin A/C is depleted, emerin is no longer localized in the nuclear membrane and instead diffuses within the endoplasmic reticulum (ER) membrane [49]. In this regard, emerin was mainly confined to the nuclear envelope in macrophages cultured on pTi but was released into the cytoplasm in macrophages cultured on TNTs despite an unchanged protein level (Fig. 5A, S7). In addition, emerin is a key modulator of actin organization, and loss of emerin from the nucleus disrupts actin dynamics [50]. When we silenced emerin with siRNA in macrophages cultured on pTi, the F/G-actin ratio was significantly reduced (Fig. 5B-E, S8). Therefore, we considered the possibility that reduced lamin A/C expression in macrophages cultured on TNTs dislocated emerin from the nuclear envelope, leading to reduced F-actin formation. When lamin A/C was overexpressed in macrophages cultured on TNTs, emerin was redirected to the nuclear envelope (Fig. 5F), and the F/G-actin ratio was increased (Fig. 4E, F). Furthermore, the increased F/G-actin ratio and inflammatory gene expression in lamin A/C-overexpressing cells cultured on TNTs were significantly reversed by emerin knockdown (Fig. 5G-I). Taken together, these data indicated that TNTs regulated the lamin A/C/emerin axis to disrupt actin-regulated inflammatory activation.

### TNTs regulated lamin A/C levels via actomyosin contractility

Recent studies have reported that changes in actomyosin contractility induced by mechanical signals are highly correlated with nuclear lamina alterations [33]; thus, we speculated that TNTs regulate lamin A/C levels by changing myosin II contractility. Immunostaining and Western blotting results (Fig. 5A-C) showed that phosphorylated myosin-II (pMLC) was notably decreased in macrophages seeded on TNTs and stimulated with LPS, suggesting that the pMLC level was influenced by the adhesive microenvironment. To further investigate how the dynamics of adhesion influenced actomyosin contractility and lamin A/C levels, we cultured macrophages on pTi and TNTs surfaces and measured the expression of target proteins over time after seeding (Fig. 5D, E). As cellular adhesion progressed on the pTi surface, pMLC progressively increased. Conversely, pMLC in macrophages cultured on TNTs appeared to slowly increase and remained at a lower level. Analysis of lamin A/C levels showed similar trends to pMLC during the adhesion process, suggesting that lamin A/C expression was potentially associated with the phosphorylation of myosin-II. To test this hypothesis, we applied blebbistatin (myosin II inhibitor) to macrophages cultured on pTi and found, as expected, that blebbistatin significantly inhibited lamin A/C expression (Fig. 5F, G). Under LPS-stimulated inflammatory conditions, blebbistatin also diminished the lamin A/C levels in macrophages cultured on pTi (Fig. 5H, I), indicating that myosin contractility might be involved in the lamin A/C-mediated regulation of inflammation. However, inhibition of myosin II activity by blebbistatin had no effect on inflammatory gene expression profiles (Fig. S9). These data demonstrated that actomyosin contractility could be partly involved in the regulation of lamin A/C levels by TNTs, and other potential mechanisms remain to be further explored.

### TNTs exhibited superior osteoimmunity under inflammatory conditions

**Fig. 6:**
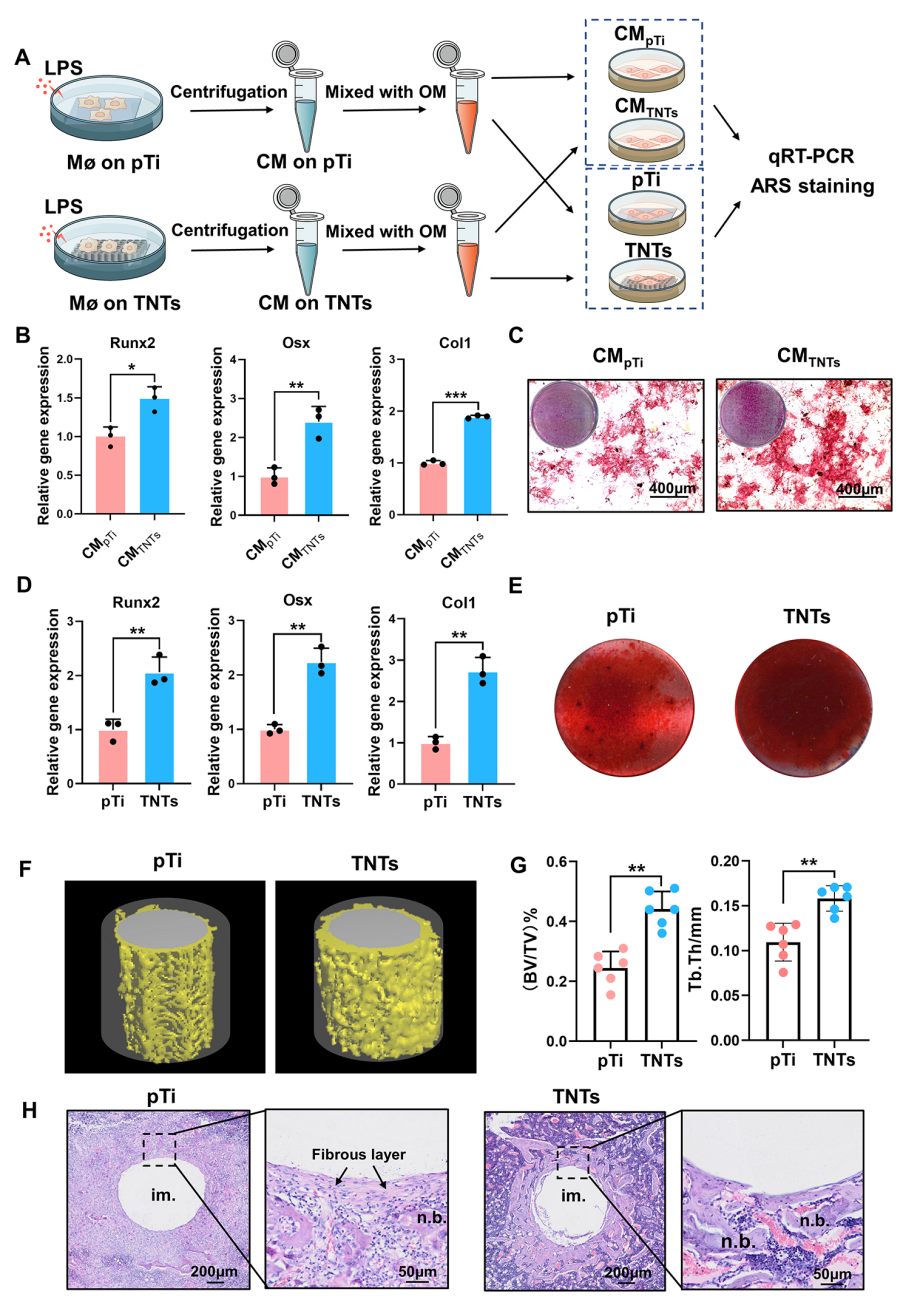
**The immunomodulatory effects of TNTs facilitated osteogenesis in vitro and in vivo under inflammatory conditions.** A) Schematic illustration of the experimental procedure. OM: osteogenic medium; CM: macrophage-conditioned medium. B-C) The expression of osteogenic genes (Runx2, Osx, and Col1) and ARS staining of mBMSCs plated in petri dishes and cultured with macrophage conditioned medium. (D, E) The expression of osteogenic genes and ARS staining of mBMSCs plated on pTi and TNTs surfaces and cultured with surface-corresponding conditioned medium. The data are presented as the mean ± SD of three biological replicates. F) Micro-CT images of 3D-reconstructed models showing the implant and bone formation in the region of interest (ROI) (grey: implant; yellow: bone tissue; semitransparent cylinder: ROI). G) Quantitative analysis of the newly formed bone volume around the pTi and TNTs implants in inflamed rats. H) Histological observation of new bone formation around the pTi and TNTs implants in inflamed rats by H&E staining (im., implant, n.b., new bone). *p < 0.05, **p < 0.01, ***p < 0.001

We further explored whether TNTs surface was capable of modulating macrophage inflammatory response to promote osseointegration under chronic inflammation. We initially investigate the effect of nanopatterning-dependent osteoimmunity on the osteogenic differentiation of mBMSCs under LPS-induced inflammatory conditions, the supernatants of RAW264.7 macrophages cultured on pTi and TNTs surfaces and stimulated with LPS were separately collected to culture mBMSCs in petri dishes. As shown in Fig. 6B, C, the mBMSCs that were cultured with TNTs-modulated macrophage conditioned medium showed higher expression of osteogenic-related genes, including Osterix (Osx), related transcription factor 2 (Runx2), and collagen I (Col1), after 7 days and higher extracellular matrix (ECM) mineralization after 21 days. Since both MSCs and macrophages accumulate at the interface of implants and surrounding bone in vivo [51], we further examined the crosstalk between these two kinds of cells on different surfaces in vitro. Under LPS-induced inflammatory conditions, TNTs exhibited higher osteoimmunomodulatory activity, showing higher levels of Osx, Runx2, and Col1 at day 7 and higher ECM mineralization at day 21 (Fig. 6D, E). Furthermore, we investigated whether nanotube patterning could affect early osseointegration under chronic inflammatory conditions in vivo. Rats were intraperitoneally injected with LPS to establish an inflammation model, and the rats showed significant neonatal bone loss around implants compared with the control rats (Fig. S10). To evaluate the osseointegration of pTi and TNTs implants in the inflamed rats, the implanted samples were harvested after 14 days for further research. In the three-dimensional (3D) reconstructed microcomputed tomography (CT) images (Fig. 6F), TNTs displayed a higher degree of new bone formation surrounding the implant in inflammatory microenvironments, which was further evidenced by quantitative micro-CT analysis, including higher bone volume fraction (BV/TV) and trabecular thickness (Tb. Th) (Fig. 6G). Histochemically, haematoxylin-eosin (H&E) staining (Fig. 6H) showed a severe inflammatory response, a thick fibrous layer, and reduced bone mass in the pTi group. In contrast, milder inflammatory reactions and more connected neonatal bone were observed around the TNTs. Collectively, these results suggested that TNTs effectively modulated the immune response of macrophages to facilitate osseointegration under systemic chronic inflammation.

## Discussion

Nanopatterning of implant surfaces is recognized as an approach for modulating the inflammatory-immune microenvironment to facilitate osseointegration [17]; however, the mechanisms underlying immune response regulation by nanopatterning are still not fully understood. In the current study, we demonstrated that titania nanotube patterning decreased the macrophage inflammatory response via lamin A/C-mediated nuclear mechanotransduction. Specifically, TNTs inhibited lamin A/C expression, which dislocated emerin from the nucleus, thus suppressing actin polymerization. The decrease in F-actin levels impaired the nuclear translocation of the actin-dependent transcription cofactor MRTF-A, leading to inhibition of the macrophage inflammatory response. Additionally, we preliminarily elucidated that decreased myosin contractility is associated with the downregulation of lamin A/C expression by TNTs. Finally, we showed that TNTs exhibited superior osteoimmunomodulation for enhancing mBMSC osteogenic differentiation in vitro and promoting osseointegration in vivo under inflammatory conditions.

Although the survival rate of implants has been reported to be over 90%, compromised bone conditions endanger high success rates [52, 53]. The main concern is implicated in diabetes, aging, osteoporosis, etc. in which the cellular microenvironment was characterized as chronic inflammation commonly, hampering bone healing around implants [7–10]. Under these chronic conditions, macrophages failed to complete their dynamic transition to anti-inflammatory phenotype, instead of persistent presence as pro-inflammatory phenotype, leading to high levels of inflammatory cytokines secretion, such as IL-1β, IL-6, and so on. These proinflammatory cytokines inhibit osteogenic differentiation of stem cells, leading to abnormal new bone formation around implants in vivo [54–56]. The methods for enhancing implant osseointegration under impaired bone conditions remain far from adequately developed [57]. In our study, we found that titania nanotubes tuned the macrophage response to LPS, resulting in significantly reduced inflammatory gene expression and cytokine secretion and modulating the local immune microenvironment, which subsequently facilitated the osteogenic differentiation of mBMSCs in vitro and osseointegration in vivo under LPS-induced inflammatory conditions. Therefore, TNTs are a promising nanotopography for manipulating osteoimmunity in favour of osseointegration under chronic inflammation; these results show the importance of nanotopography-mediated osteoimmuno-modulation in bone regeneration.

A number of studies have reported the notable effects of surface nanopatterning on macrophage function [22]; however, the underlying mechanisms remain unclear. In this study, we identified lamin A/C as a key mechanoresponsive molecule for sensing nanotopography and controlling macrophage inflammatory response. We found that lamin A/C in macrophages is associated with surface nanopatterning, showing significantly reduced lamin A/C protein levels in macrophages cultured on titania nanotubes compared with those cultured on the smooth titanium surface. In addition, we showed that lamin A/C modulated macrophage inflammatory response, and depletion of lamin A/C was associated with decreased proinflammatory gene expression after LPS stimulation, which is consistent with a previous study [37]. Impressively, the TNTs-induced downregulation of inflammatory gene expression in RAW264.7 macrophages could be reversed by transfection with a lamin A/C-overexpressing lentivirus. Together, these data showed that modulation of lamin A/C expression by TNTs tunes the macrophage inflammatory response to LPS. In previous studies, lamin A/C has been implicated in mechanotransduction in several cells [58], including fibroblasts [59] and mesenchymal stem cells [28], among others. The present study extends these observations, showing lamin A/C plays a key role in macrophage mechanotransduction.

The above results inspired us to investigate how lamin A/C regulates the macrophage inflammatory response. LPS-stimulated proinflammatory macrophage activation requires actin polymerization and subsequent nuclear translocation of the actin-dependent transcription cofactor MRTF-A, which leads to the expression of inflammatory genes [24]. Interestingly, actin stress fibres are indirectly attached to lamin A/C, and fewer stress fibres are observed in human mesenchymal stromal cells (hMSCs) after knockdown of lamin A/C [47]. Thus, we hypothesized that lamin A/C may depend on the actin-related inflammatory signalling pathway to regulate macrophage inflammatory response. In this study, we found that lamin A/C expression changed earlier than actin polymerization during LPS stimulation. In detail, lamin A/C significantly increased after 1 h of LPS treatment, while the F/G-actin ratio and nuclear translocation of MRTF-A increased only after 6 h of LPS treatment [24]. Moreover, the overexpression of lamin A/C in macrophages cultured on TNTs promoted F-actin formation and nuclear translocation of MRTF-A. Notably, when actin polymerization was inhibited by cytochalasin D, the expression levels of inflammatory markers in lamin A/C-overexpressing cells were significantly suppressed. These data showed for the first time that lamin A/C in macrophages mediates the regulation of the macrophage inflammatory response by TNTs via the actin-regulated inflammatory pathway, suggesting the synergistic effect of the nucleoskeleton and cytoskeleton in modulating macrophage function.

As our research progressed, we wondered how lamin A/C modulated actin organization. Emerin, which is an inner nuclear membrane protein, requires lamin A/C for proper localization. In the absence of lamin A/C, emerin is no longer localized to the nuclear membrane [50]. In this regard, we found that emerin was distributed to the cytoplasm in macrophages cultured on TNTs and was relocated to the nucleus after lamin A/C overexpression, indicating that the lamin A/C level in macrophages cultured on TNTs determines the spatial distribution of emerin. In addition, emerin is an actin pointed-end capping protein that regulates actin polymerization [60]. Our results showed that knockdown of emerin resulted in reduced F-actin formation. Importantly, we showed that in lamin A/C-overexpressing macrophages, when we reduced the nuclear localization of emerin, the F/G-actin ratio was decreased, and the inflammatory response was suppressed. Therefore, we confirmed that emerin was required for lamin A/C to mediate actin-regulated inflammation. Similarly, Lmna^N195K/N195K^ mutant embryonic fibroblasts, which are related to dilated cardiomyopathy, have impaired actin polymerization due to emerin delocalization from the nucleus, which affects cardiac development and function [50]. To the best of our knowledge, this study is the first to confirm the effect of the lamin A/C-emerin axis on mediating macrophage inflammatory response.

Although an association was demonstrated between nanopatterning and lamin A/C levels in macrophages, the specific mechanism requires further investigation. Cells are well known to respond to external physical cues by changing their cytoskeletal structures [26]. Moreover, the cytoskeleton propagates mechanical stresses to the nucleus, affecting its molecular expression and mechanical properties [27]. For example, increases in matrix stiffness upregulates myosin II contractility, leading to increased lamin A/C levels in mesenchymal stem cells (MSCs)[33]. In this study, we found that inhibition of myosin-II-generated tension was partly involved in the downregulation of lamin A/C by TNTs. We showed that changes in lamin A/C levels were associated with cell adhesion, paralleling changes in pMLC. When myosin II contractility was inhibited by blebbistatin, lamin A/C levels were significantly reduced in macrophages. However, lamin A/C-mediated inflammatory gene expression was not altered by treatment with pharmacological inhibitors of myosin, which is consistent with Jain’s findings [24]. This indicated that the mechanism by which lamin A/C is regulated by TNTs is complex and not limited to the effects of cell contractility, and this mechanism deserves to be thoroughly investigated in our future studies. Nonetheless, these results showed the importance of nanopatterning for regulating lamin A/C expression in order to control macrophage inflammatory response, and these effects cannot be achieved by the administration of a pharmacological inhibitor alone.

## Conclusions

In summary, this study demonstrated that titania nanotubes inhibited the macrophage inflammatory response by reducing lamin A/C expression. Mechanistically, reduced lamin A/C expression in macrophages cultured on TNTs suppressed actin-regulated MRTF-A-SRF complex activity by reducing the nuclear localization of emerin, therefore decreasing the inflammatory response. Finally, we showed that nanoscale TNTs coatings on implants exerted superior osteoimmunomodulatory effects to promote osseointegration under inflammatory conditions. These outcomes identify lamin A/C as a key mechanosensitive molecule for controlling macrophage inflammatory responses, which has the potential to inform the development of immunomodulatory surface topographies in the future.

## Electronic supplementary material

Below is the link to the electronic supplementary material.

Supplementary Material 1

## Data Availability

All data generated or analysed during this study are included in this published article.
