# Supplementary material for: Nanotube patterning reduces macrophage inflammatory response via nuclear mechanotransduction
Source: J Nanobiotechnology. 2023 Jul 19;21:229. doi: 10.1186/s12951-023-01912-4 (PMC10354937; doi:10.1186/s12951-023-01912-4)
Supplement: Supplementary file 1 — Supplementary Material 1 [file 12951_2023_1912_MOESM1_ESM.docx]

Supporting Information

**Nanotube patterning reduces macrophage inflammatory response via nuclear mechanotransduction**

Yiru Fu^1^, Zheng Jing^1,2,3^, Tao Chen^1^, Xinxin Xu^1^, Xu Wang^1^, Mingxing Ren^1^, Yanqiu Wu^1^, Tianli Wu^1^, Yuzhou Li^1,2,3^, He Zhang^1,2,3^*, Ping Ji^1,2,3^*, Sheng Yang^1,2,3^*

1.College of Stomatology, Chongqing Medical University, Chongqing, China.

2.Chongqing Key Laboratory of Oral Diseases and Biomedical Sciences, Chongqing, China.

3.Chongqing Municipal Key Laboratory of Oral Biomedical Engineering of Higher Education, Chongqing, China.

Corresponding authors:

Dr. He Zhang, College of Stomatology, Chongqing Medical University. 426#Songshibei Road, Yubei District, Chongqing 401147, China.

1. mail address: kqzhanghe@hospital.cqmu.edu.cn

Dr. Ping Ji, College of Stomatology, Chongqing Medical University. 426#Songshibei Road, Yubei District, Chongqing 401147, China.

E-mail address: jiping@hospital.cqmu.edu.cn

Dr. Sheng Yang, College of Stomatology, Chongqing Medical University. 426#Songshibei Road, Yubei District, Chongqing 401147, China.

Tel.: +86-23-8860-2351; Fax: +86-023-8886-0085;

E-mail address: [ysdentist@hospital.cqmu.edu.cn](mailto:ysdentist@hospital.cqmu.edu.cn)


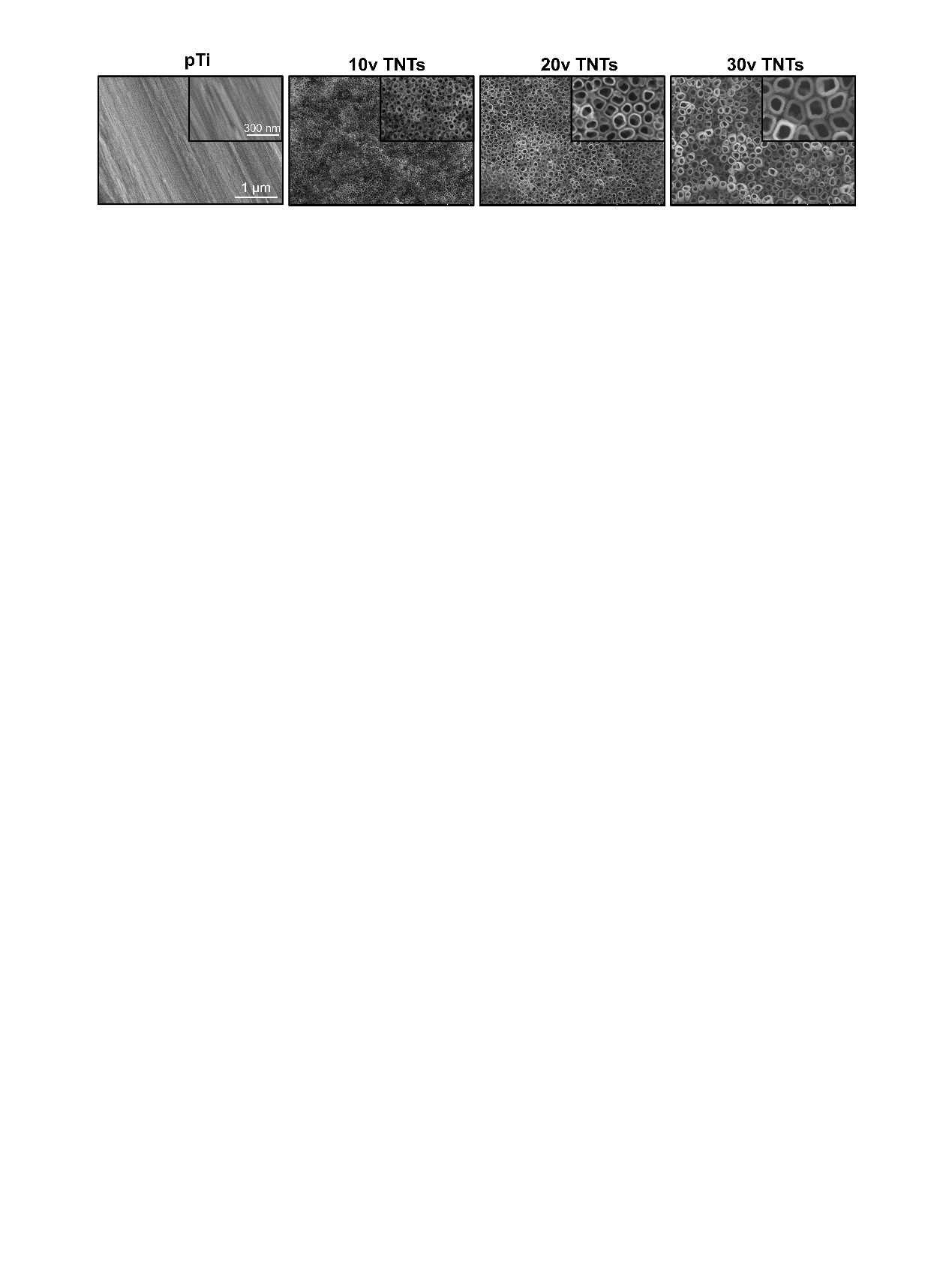


**Fig S1.** SEM images of different Ti substrates.


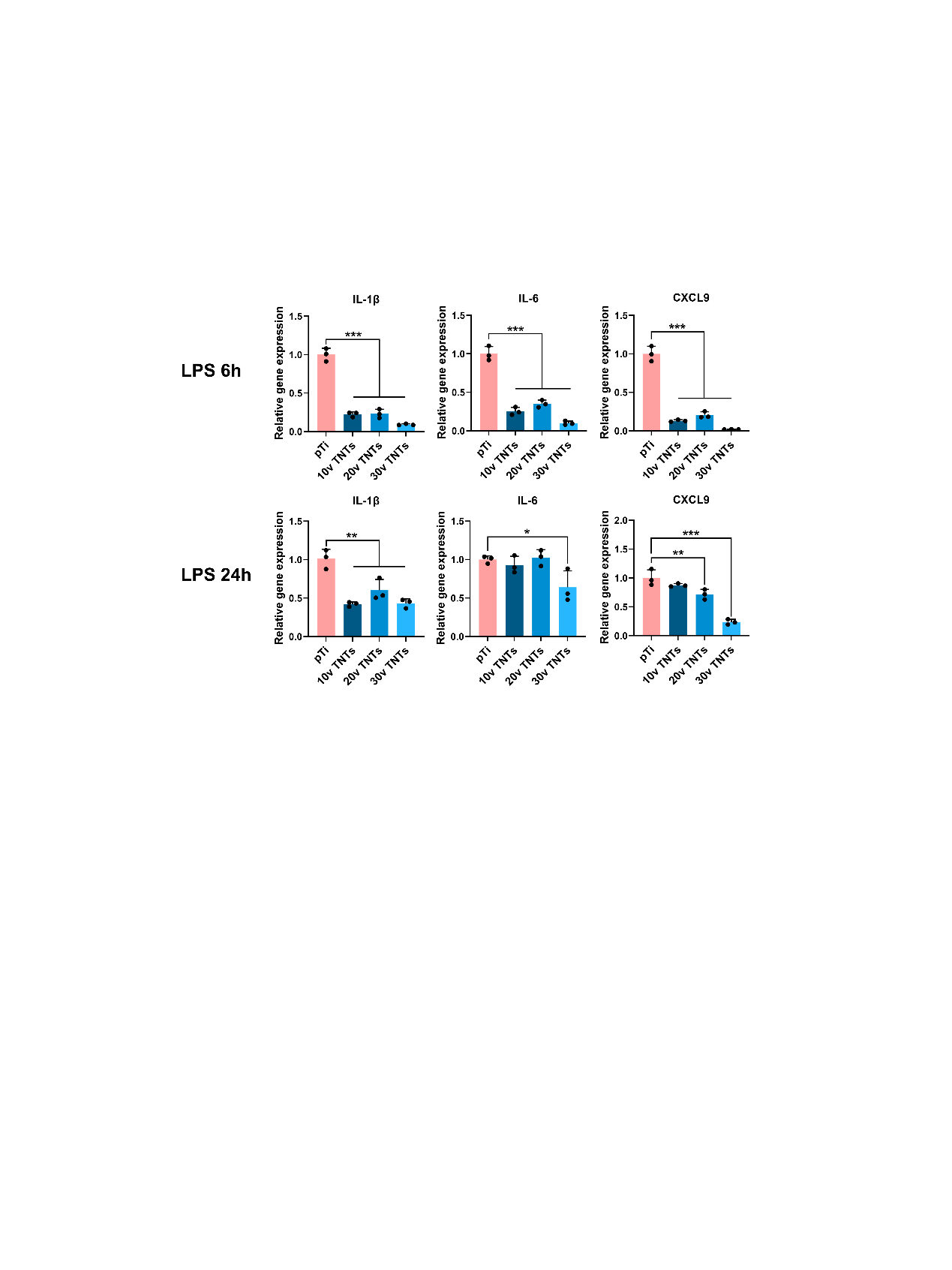


**Fig S2.** The expression of inflammatory genes (IL-1β, IL-6, and CXCL9) in macrophages cultured on different Ti substrates and stimulated with LPS for 6 h or 24 hours. The data are presented as the mean ± SD of three biological replicates. *p<0.05, **p<0.01, ***p<0.001.


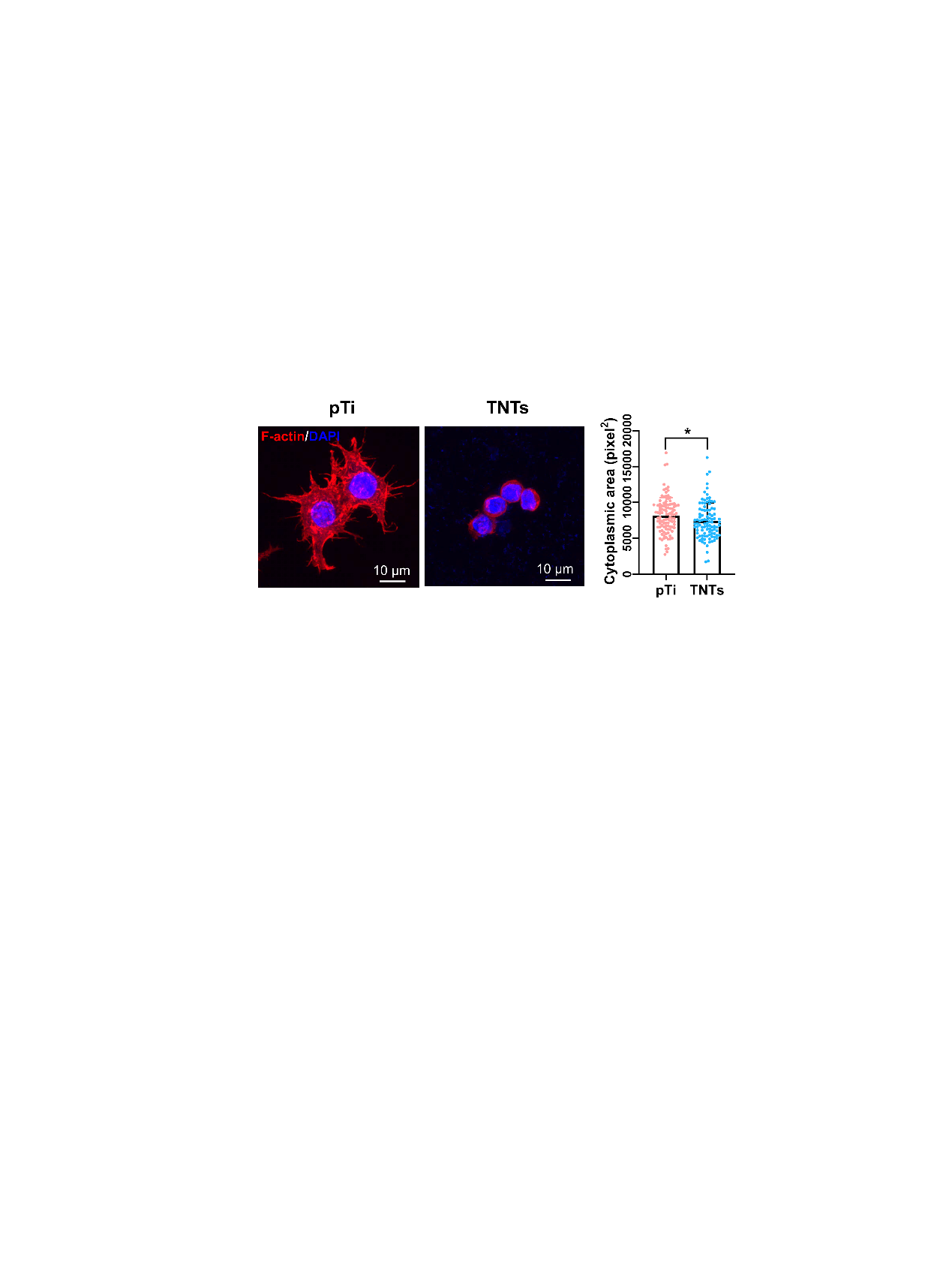


**Fig S3.** Representative images of F-actin and DAPI staining in macrophages cultured on pTi and TNTs and stimulated with LPS for 6 hours, and quantification of the cytoplasmic area. The data were pooled from 98-104 single cells in at least 21-30 randomly chosen fields. *p<0.05.


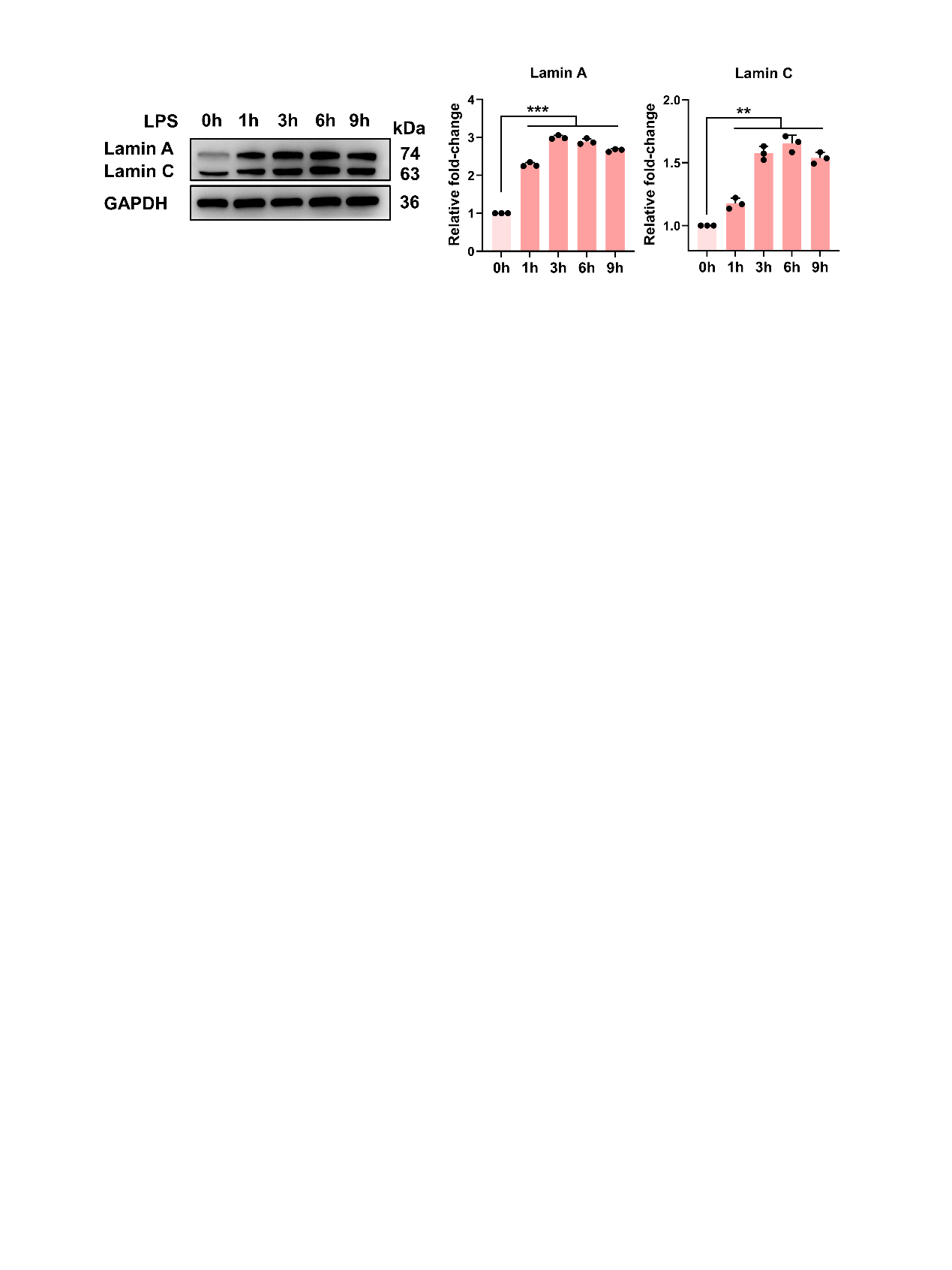


**Fig S4.** Western blotting analysis of lamin A/C expression in macrophages cultured on pTi before and after LPS treatment for 1, 3, 6, and 9 hours. The data are presented as the mean ± SD of three biological replicates. **p<0.01, ***p<0.001.


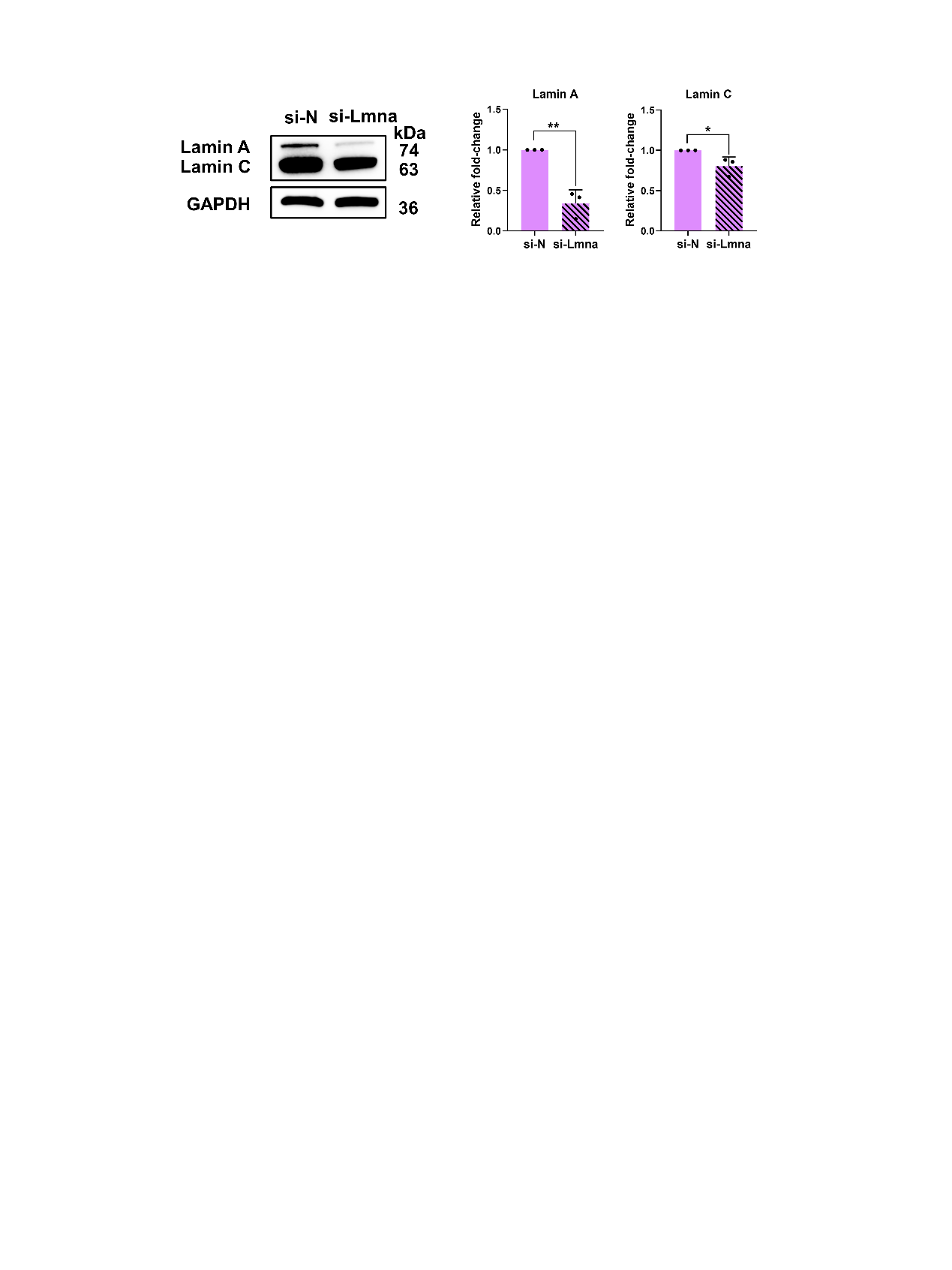


**Fig S5.** Western blotting analysis of lamin A/C expression in macrophages treated with Lmna siRNA or nontargeting control for 24 hours. The data are presented as the mean ± SD of three biological replicates. *p<0.05, **p<0.01.


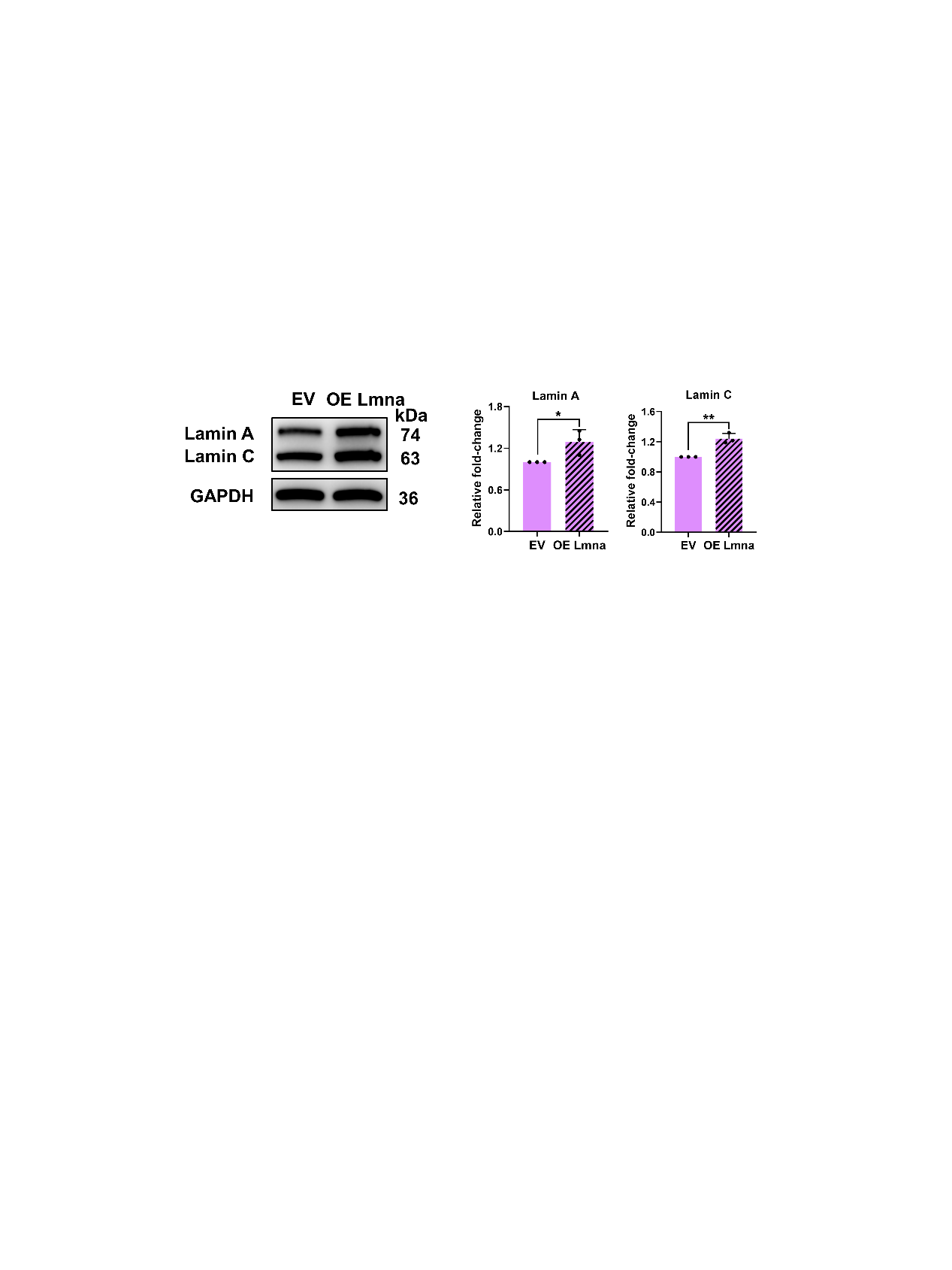


**Fig S6.** Western blotting analysis of lamin A/C expression in lamin A/C-overexpressing macrophages and lentiviral vector-expressing control cells. The data are presented as the mean ± SD of three biological replicates. *p<0.05, **p<0.01.


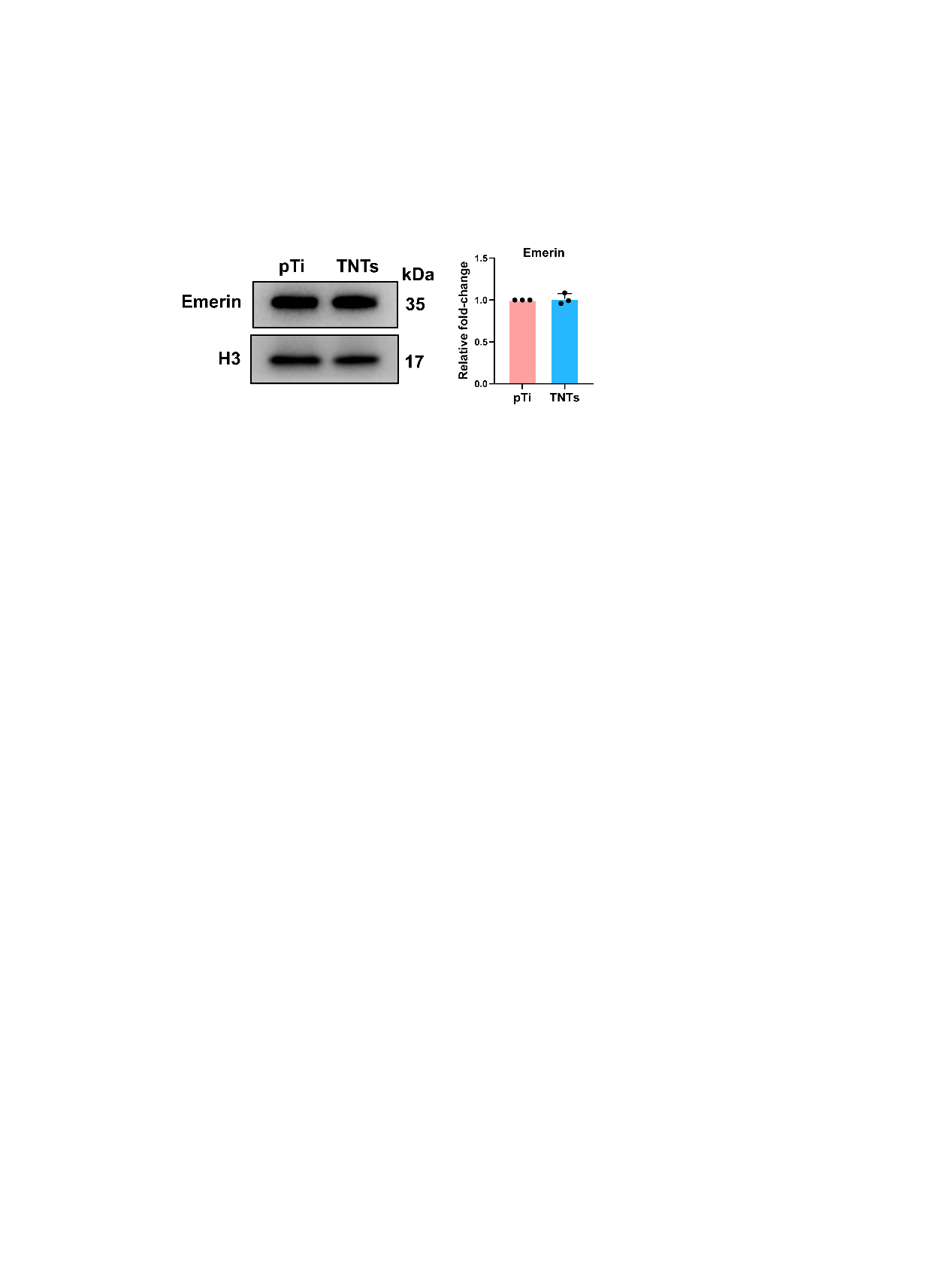


**Fig S7.** The total amount of emerin in macrophages cultured on pTi and TNTs surfaces after 6 hours of LPS treatment. The data are presented as the mean ± SD of three biological replicates.


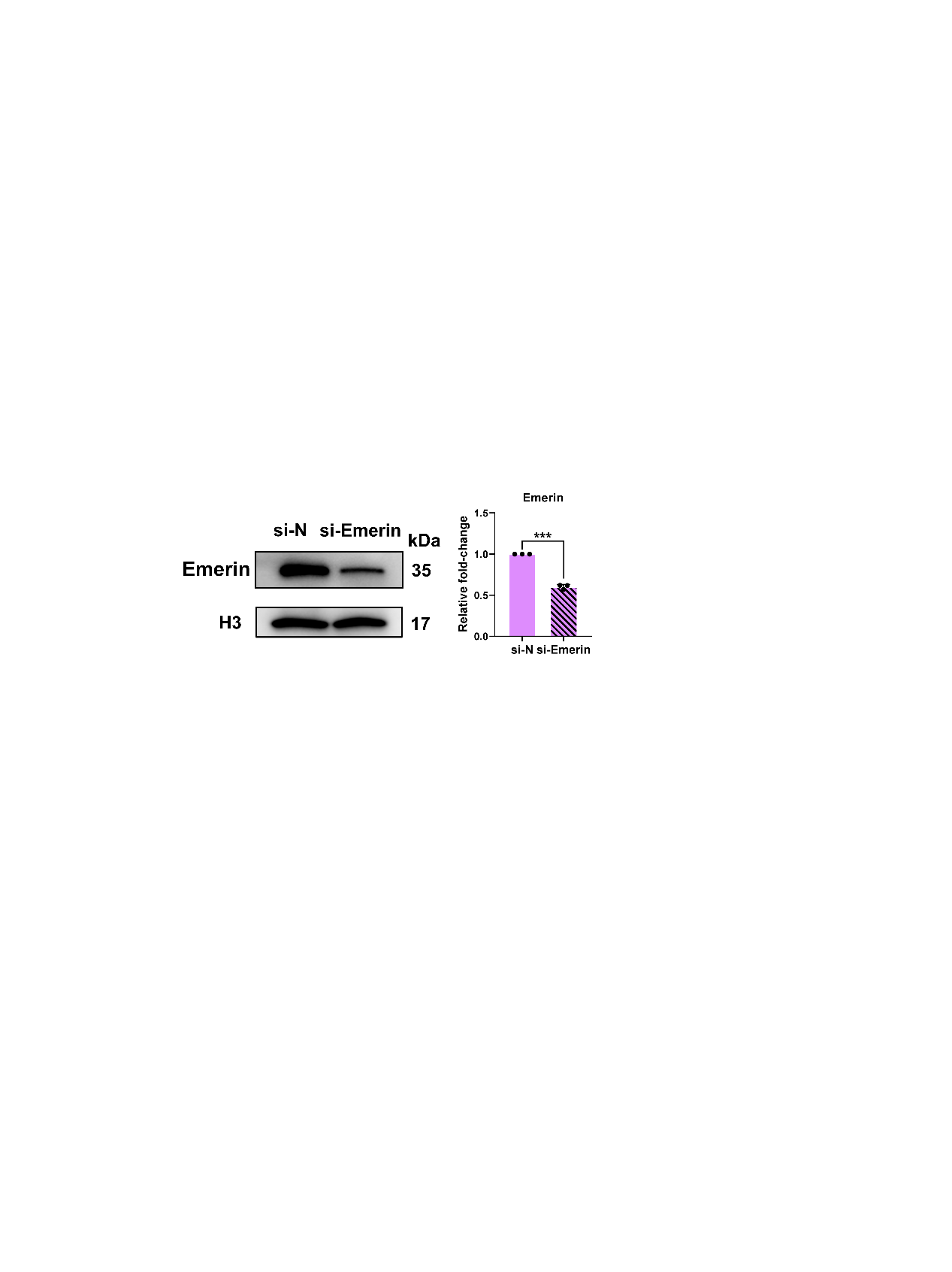


**Fig S8.** Western blotting analysis of emerin expression in macrophages treated with emerin siRNA or nontargeting control for 24 hours. The data are presented as the mean ± SD of three biological replicates. ***p<0.001.


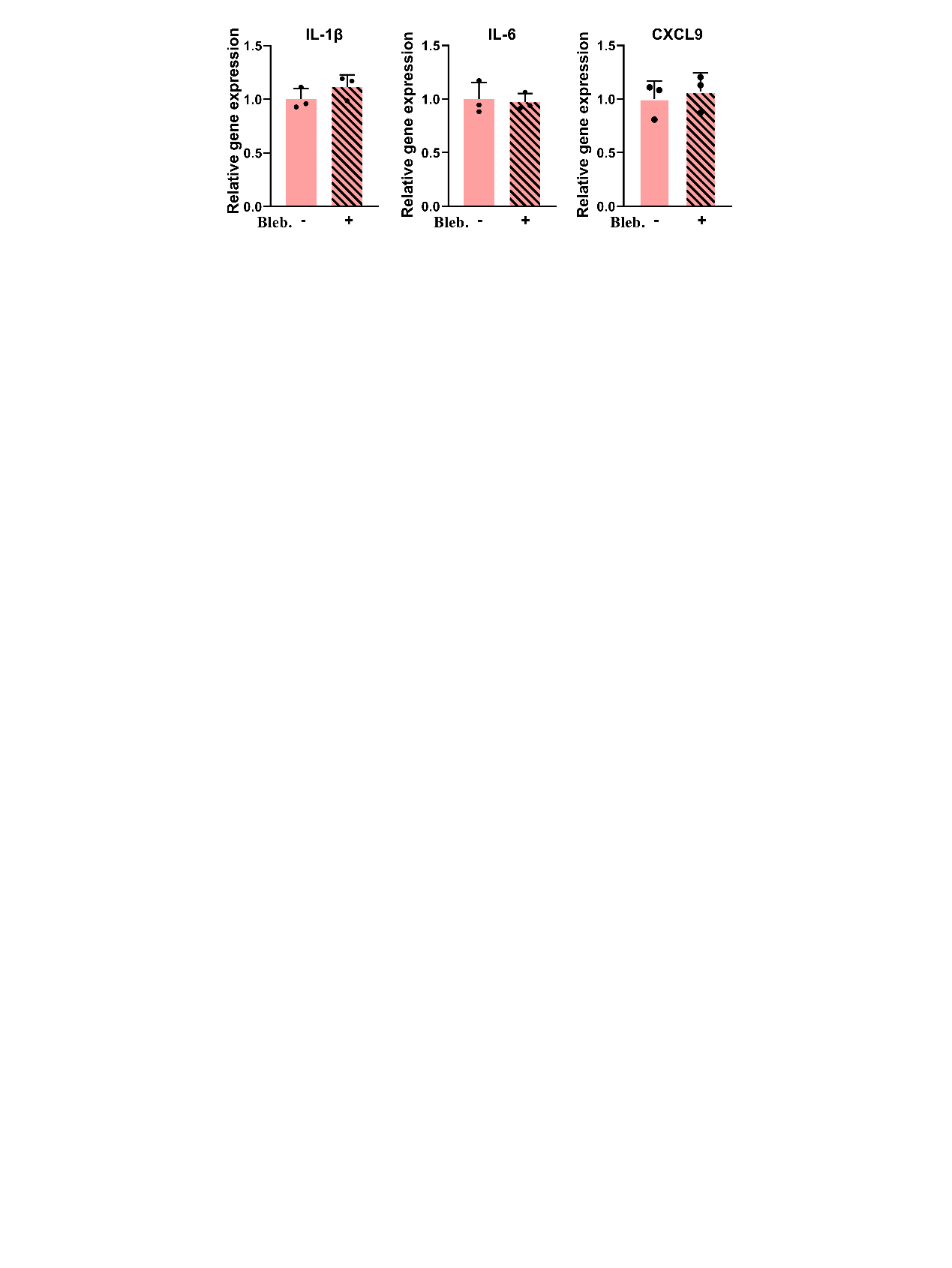


**Fig S9.** The expression of inflammatory genes (IL-1β, IL-6, and CXCL9) in LPS-treated macrophages cultured on pTi and blebbistatin (Bleb.) + LPS-treated macrophages cultured on pTi. The data are presented as the mean ± SD of three biological replicates.


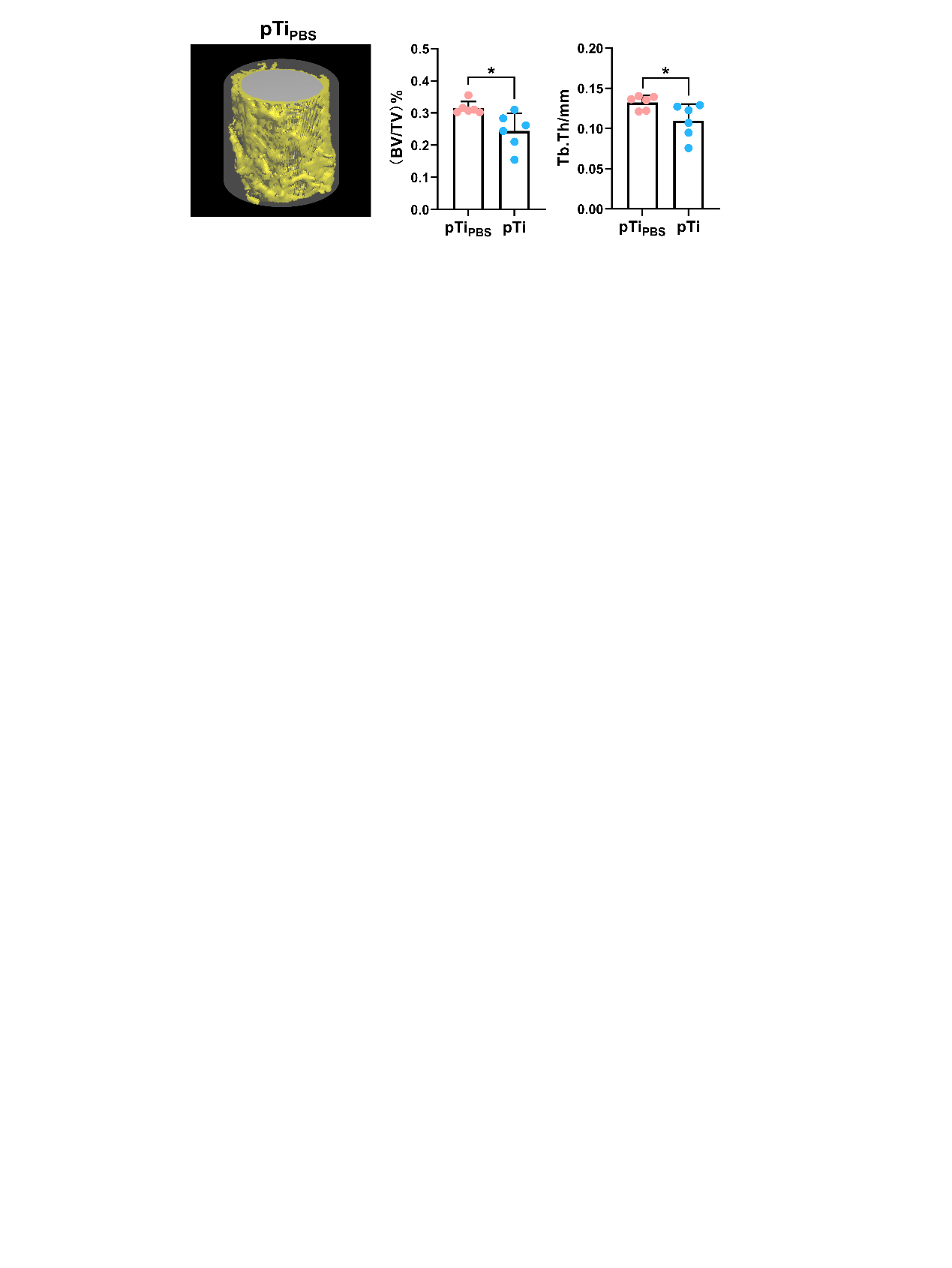


**Fig S10.** 3D reconstructed micro-CT images of pTi rods implanted in rats that were injected with PBS (grey: implant; yellow: bone tissue; semitransparent cylinder: ROI). Quantitative analysis of the newly formed bone volume around the pTi implants in normal rats and inflamed rats. The data are presented as the mean ± SD of six biological replicates. *p<0.05.

| **Table S1. Primers used for real-time PCR** | | | |
| --- | --- | --- | --- |
| **Gene** | **Forward primer sequence (5'-3')** | **Reverse primer sequence (5'-3')** |  |
| **IL-1β** | TTGAAGTTGACGGACCCCA | GAGTGATACTGCCTGCCTGAAG |  |
| **IL-6** | GTTGCCTTCTTGGGACTGATG | TTGGGAGTGGTATCCTCTGTGA |  |
| **CXCL9** | TCATTGCTACACTGAAGAACGGAG | CCTTGAACGACGACGACTTTG |  |
| **Runx2** | CGGGAACCAAGAAGGCACA | AGGCGGGACACCTACTCTCATA |  |
| **Osx** | GCCTACTTACCCGTCTGACTTTG | TGCCCACTATTGCCAACTGC |  |
| **Col1** | GGCTCTAGAGGTGAACGTGG | CACCAGGGGCACCATTAACT |  |
| **GAPDH** | TGAGGTGACCGCATCTTCTTG | TGGTAACCAGGCGTCCGATA |  |

Table S2

| Antibodies for western blot | Source | Dilution |
| --- | --- | --- |
| Lamin A/C Mouse antibody | Cell Signaling Technology | 1:1000 |
| GAPDH Mouse antibody | Proteintech | 1:1000 |
| Lamin B1 Rabbit antibody | Abcam | 1:1000 |
| Histone H3 Rabbit antibody | Cell Signaling Technology | 1:1000 |
| MRTF-A Rabbit antibody | Novus | 1:1000 |
| Emerin Rabbit antibody | Cell Signaling Technology | 1:1000 |
| Phospho-myosin light chain 2 (Ser19) (pMLC) Rabbit antibody | Cell Signaling Technology | 1:1000 |
| HRP-conjugated Affinipure Goat Anti-Mouse IgG(H+L) | Proteintech | 1:5000 |
| HRP-conjugated Affinipure Goat Anti-Rabbit IgG(H+L) | Proteintech | 1:5000 |

Table S3

| Antibodies for immunostaining | Source | Dilution |
| --- | --- | --- |
| Emerin Rabbit antibody | Cell Signaling Technology | 1:400 |
| Lamin A/C Mouse antibody | Cell Signaling Technology | 1:200 |
| Phospho-myosin light chain 2 (Ser19) (pMLC) Rabbit antibody | Cell Signaling Technology | 1:50 |
| Alexa Fluor™ 488 donkey anti-rabbit IgG (H+L) | Invitrogen | 1:300 |
| Alexa Fluor™ 647 donkey anti-mouse IgG (H+L) | Invitrogen | 1:300 |
